# Origin of the Unusual Ground-State Spin *S* = 9 in a Cr_10_ Single-Molecule Magnet

**DOI:** 10.1021/jacs.2c05453

**Published:** 2022-06-27

**Authors:** Javier Rubín, Ana Arauzo, Elena Bartolomé, Francesco Sedona, Marzio Rancan, Lidia Armelao, Javier Luzón, Tatiana Guidi, Elena Garlatti, Fabrice Wilhelm, Andrei Rogalev, Andreas Amann, Stefano Spagna, Juan Bartolomé, Fernando Bartolomé

**Affiliations:** † Instituto de Nanociencia y Materiales de Aragón (INMA), CSIC-Universidad de Zaragoza, 50009 Zaragoza, Spain; ‡ Departamento de Ciencia y Tecnología de Materiales y Fluidos, Universidad de Zaragoza, 50018 Zaragoza, Spain; § Servicio de Medidas Físicas, Universidad de Zaragoza, Pedro Cerbuna 12, 50009 Zaragoza, Spain; ∥ Departamento de Física de la Materia Condensada, Universidad de Zaragoza, 50009 Zaragoza, Spain; ⊥ Escola Universitària Salesiana de Sarrià (EUSS), Passeig Sant Joan Bosco 74, 08017 Barcelona, Spain; # Dipartimento di Scienze Chimiche, Università di Padova, Via Marzolo 1, 35131 Padova, Italy; ∇ Institute of Condensed Matter Chemistry and Technologies for Energy (ICMATE), National Research Council (CNR), c/o Department of Chemistry, University of Padova, via F. Marzolo 1, 35131 Padova, Italy; ○ Department of Chemical Sciences and Materials Technologies (DSCTM), National Research Council (CNR), Piazzale A. Moro 7, 00185 Roma, Italy; ◆ Academia General Militar, Centro Universitario de la Defensa, 50090 Zaragoza, Spain; ¶ Physics Division, School of Science and Technology, University of Camerino, Via Madonna delle Carceri 9, 62032 Camerino, MC, Italy; ⋈ ISIS Facility, Rutherford Appleton Laboratory, Chilton, Didcot OX11 0QX, Oxfordshire, U.K.; ⧓ Dipartimento di Science Matematiche, Fisiche e Informatiche, Università di Parma, Parco Area delle Scienze 7/A, 43124 Parma, Italy; ⧖ ESRF − The European Synchrotron Radiation Facility, 71 Avenue des Martyrs CS40220, F-38043 Grenoble Cedex 09, France; ● Quantum Design Inc., San Diego, California 92121, United States

## Abstract

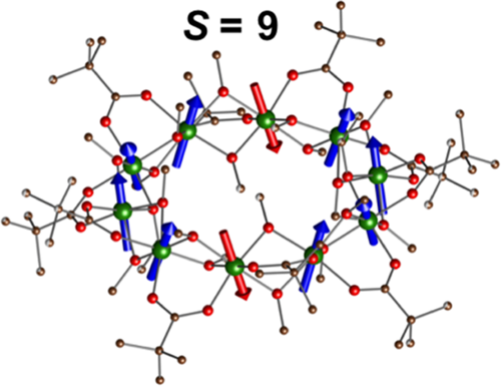

The molecular wheel [Cr_10_(OMe)_20_(O_2_CCMe_3_)_10_], abbreviated {Cr_10_}, with an unusual intermediate total spin *S* = 9 and non-negligible cluster anisotropy, *D*/*k*_B_ = −0.045(2) K, is a rare case among wheels based on an even number of 3d-metals, which usually present an antiferromagnetic (AF) ground state (*S* = 0). Herein, we unveil the origin of such a behavior. Angular magnetometry measurements performed on a single crystal confirmed the axial anisotropic behavior of {Cr_10_}. For powder samples, the temperature dependence of the susceptibility plotted as χ*T*(*T*) showed an overall ferromagnetic (FM) behavior down to 1.8 K, whereas the magnetization curve *M*(*H*) did not saturate at the expected 30 μ_B_/fu for 10 FM coupled 3/2 spin Cr^3+^ ions, but to a much lower value, corresponding to *S* = 9. In addition, the X-ray magnetic circular dichroism (XMCD) measured at high magnetic field (170 kOe) and 7.5 K showed the polarization of the cluster moment up to 23 μ_B_/fu. The magnetic results can be rationalized within a model, including the cluster anisotropy, in which the {Cr_10_} wheel is formed by two semiwheels, each with four Cr^3+^ spins FM coupled (*J*_FM_/*k*_B_ = 2.0 K), separated by two Cr^3+^ ions AF coupled asymmetrically (*J*_23_/*k*_B_ = *J*_78_/*k*_B_ = −2.0 K; *J*_34_/*k*_B_ = *J*_89_/*k*_B_ = −0.25 K). Inelastic neutron scattering and heat capacity allowed us to confirm this model leading to the *S* = 9 ground state and first excited *S* = 8. Single-molecule magnet behavior with an activation energy of *U*/*k*_B_ = 4.0(5) K in the absence of applied field was observed through ac susceptibility measurements down to 0.1 K. The intriguing magnetic behavior of {Cr_10_} arises from the detailed asymmetry in the molecule interactions produced by small-angle distortions in the angles of the Cr–O–Cr alkoxy bridges coupling the Cr^3+^ ions, as demonstrated by *ab initio* and density functional theory calculations, while the cluster anisotropy can be correlated to the single-ion anisotropies calculated for each Cr^3+^ ion in the wheel.

## Introduction

Magnetic molecular wheels are a subclass of molecular magnets that have received considerable attention for their intrinsic magnetic properties, as benchmark systems for the investigation of macroscopic quantum coherent phenomena, and in view of their possible application in quantum information processing.^1−3^ The planar and high-symmetry geometry of cyclic molecules makes them in addition attractive for deposition onto substrates, a critical step toward device fabrication.^4,5^

Magnetic wheels made of different 3d transition metals (Cr, Ni, Cu, V, Mo, Mn, Fe, etc.) have been extensively investigated.^6,7^ For example, ferric wheels of different nuclearity (Fe_6_,^8^ Fe_10_,^9−11^ Fe_12_,^12^ Fe_18_^13^) have been reported presenting dominant antiferromagnetic (AF) coupling between the Fe^3+^ ions, with *S*_i_ = 5/2 spin, and a ground state of total spin *S* = 0. In contrast, single-molecule magnet (SMM) behavior was found for noncyclic clusters Fe_8_ with a ground state *S* = 10 and activation barrier for spin reversal *U*/*k*_B_ = 24.5 K ^14^ and in Fe_4_ propellers (with *S* = 5 and *U*/*k*_B_ = 3.5 ^15^ to 15.6 K ^16^), for which quantum tunneling of the magnetization (QTM) between the states ±*m* was observed. A Ni_12_ cyclic cluster showing ferromagnetically (FM) coupled *S*_i_ = 1 Ni spins giving rise to a high spin *S* = 12 ground state has been reported.^17^ The presence of two different nearest-neighbor FM interactions, and an AF nearest-neighbor interaction could only be assessed by means of inelastic neutron scattering experiments (INS), showing the relevance of this type of experiments in the resolution of the intracluster interactions. The SMM behavior corresponded to *U*/*k*_B_ = 9.6 K.

In particular, Cr^3+^ wheels have been intensively studied. The best well-known molecule of this family and precursor of other cyclic systems is {Cr_8_},^18,19^ characterized by a perfect AF coupling between each of the eight Cr^3+^ ions with spin *S*_i_ = 3/2, leading to a total *S* = 0 ground state. Four-dimensional INS allowed to directly obtain the dynamic correlation functions, confirming the existence of anisotropy and the presence of *S*-mixing, while demonstrating^20^ that the low-temperature dynamics of {Cr_8_} is not determined by coherent Néel vector tunneling, as it had been proposed earlier.^21^

Starting from the synthesis of that molecule, a whole series of other homometallic^22,23^ and heterometallic Cr-based cages were engineered.^24−28^ The heterometallic counterparts were used to compare the spin dynamics of AF closed {Cr_8_} and open {Cr_8_Zn} rings,^29^ the latter displaying quantum oscillations of the total spin under an applied field.^30,31^ Finite-size effects were also observed on the local magnetization of open rings.^32,33^ Furthermore, heterometallic {Cr_7_Ni}^34,35^ rings containing seven Cr^3+^ ions and one Ni^2+^ ion AF coupled, with an *S* = 1/2 ground state, have been considered very carefully, as possible candidates for qubits.^2,4^

An interesting case is the family of {Cr_10_} wheels of general formula [Cr_10_(OR)_20_(O_2_CR′)_10_] reported by Low et al.,^36^ whose magnetic properties strongly depend on the OR and O_2_CR′ ligands. Susceptibility measurements revealed that those members of the family with ethoxy group ligands (Et) interconnecting the Cr^3+^ ions exhibited AF behavior (*S* = 0). The exchange constants could be readily obtained from the Curie–Weiss law. In contrast, when the interconnecting ligand contains a methyl group, giving rise to the methoxy group OMe, the Cr^3+^ spins couple ferromagnetically, as determined by the Curie–Weiss positive constant, though an AF coupling contribution, observed as a downturn in χ*T*, appeared in the compounds with the smaller R′ groups, which was assigned to small intermolecular interactions.

In both the AF and FM wheels, the values of the interaction constants depend on the external ligand O_2_CR’. Only one of the members of this family, denoted as **5** in ref (36), carrying R = Me and the largest R′ group (CMe_3_), shows an overall FM behavior down to the lowest temperature (1.8 K), although it is peculiar, as the magnetization does not saturate at the expected 30 μ_B_/fu for 10 FM coupled 3/2 spin Cr^3+^ ions but to a much lower value, indicating a ground state different from *S* = 15. Electron paramagnetic resonance (EPR) measurements were performed by Sharmin et al.^37^ for this member of the family, with applied magnetic field along the axis perpendicular to the average {Cr_10_} ring’s plane. The absorption spectrum indicated a spin *S* = 9 ground state, whose temperature dependence suggests an excited state at ca. 10 K, proposed to be a spin *S* = 10 multiplet. The single-molecule uniaxial anisotropy constant was derived to be *D*/*k*_B_ = −0.045(4) K from the spectrum taken at 7 K.

The unusual intermediate ground-state spin *S* = 9 of this wheel has remained without explanation for a long time, apart from the assumption of displaying both FM and AF exchange interactions in the wheel. The presence of more than one value of exchange constants in this family of compounds had already been suggested by Low et al.^36^ in the wheel denoted as **1** with all FM interactions. We propose a model of interactions along the full wheel, which is supported by both *ab initio*, DFT calculations and experimental results, and shows a correlation of structural data and interaction parameters all.

**Figure 1:**
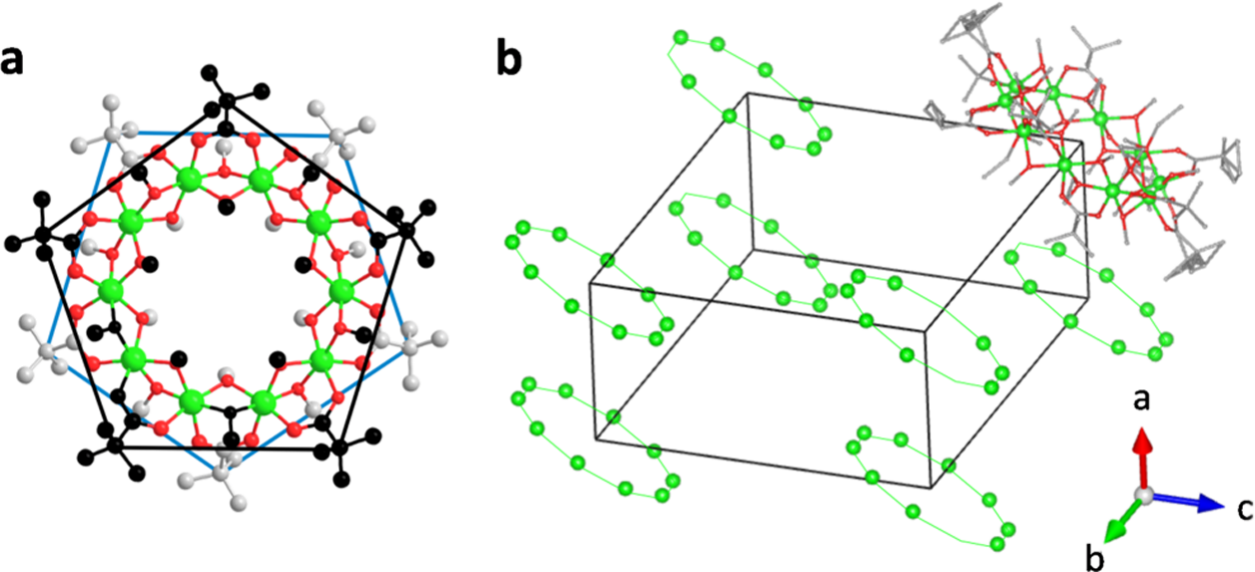
Structure of {Cr_10_(OMe)_20_(O_2_CCMe_3_)_10_} wheel. (a) Local D_5d_ quasi-symmetry of carboxylate ligands has been outlined by lines. Color code: Cr green, O red, C black/gray atoms above/below the plane of metal atoms; (b) {Cr_10_} wheels are tilted with respect to the stacking direction along the *a*-axis.

In the present work, we investigate in-depth the magnetic properties of wheel **5** (Figure 1), from now on simply called {Cr_10_} to relate them to a model of exchange interactions and single-ion anisotropies along the 10 Cr^3+^ ions of the full wheel. This model gives a consistent interpretation of the origin of the *S* = 9 ground state.

In the next sections, we will show angular magnetometry on a single crystal (SC), which has allowed us to directly observe the wheel’s magnetic anisotropy, in agreement with EPR data, and XANES and XMCD spectra at the Cr K-edge, which are used to determine the magnetic moment of Cr^3+^ ions. The model proposed to explain the existence of the *S* = 9 ground state is supported by low-temperature INS spectra and heat capacity measurements. Finally, the occurrence of SMM behavior, with an activation energy approximately given by *DS*_z_^2^, is shown through ac susceptibility measurements down to 0.1 K.

## Results

### Dc Magnetometry of Polycrystalline {Cr_10_}

**Figure 2:**
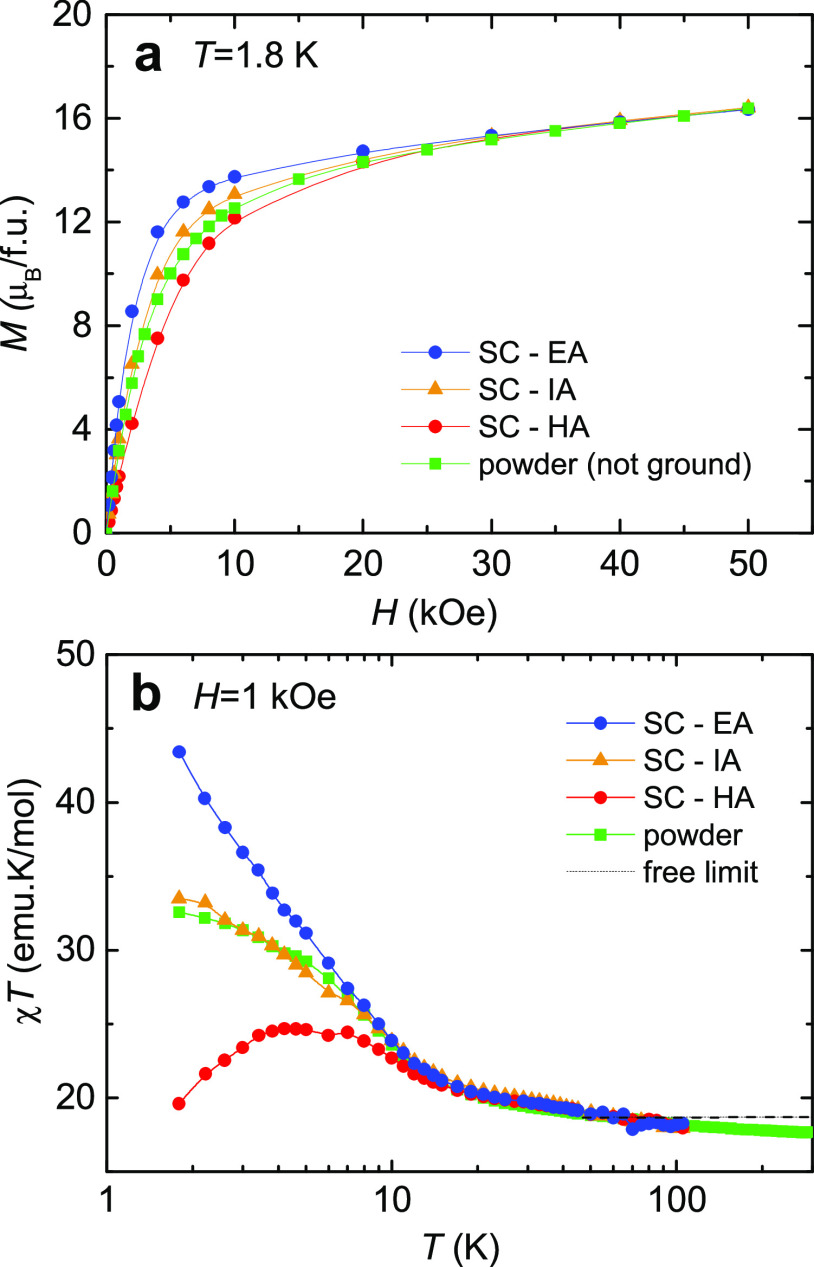
Dc magnetometry. (a) Field dependence of the magnetization, *M*(*H*), at *T* = 1.8 K for a powder sample of {Cr_10_} and SC sample measured with the applied magnetic field parallel (EA) and perpendicular (HA) to the easy axis of magnetization, and at an intermediate angle (IA); (b) temperature dependence of the susceptibility-temperature, χ*T*(*T*), for the same samples. The dotted line corresponds to the free-ion high *T* limit, χ*T*_300K_ = 18.7 emu K/mol.

The magnetization as a function of the applied field, *M*(*H*), measured for a polycrystalline sample at *T* = 1.8 K is shown in Figure 2a (green symbols). The magnetization does not saturate to the expected value of 30 μ_B_/fu for 10 uncoupled Cr^3+^ ions but reaches a much smaller value of 16.8 μ_B_/fu at the maximum applied field of *H* = 50 kOe, suggesting that the ground state is not *S* = 15 but possibly *S* = 9.

The temperature dependence of susceptibility, plotted as χ*T*(*T*), is shown in Figure 2b for a powder sample fixed in cotton wool to prevent the orientation of the grains. At high temperature, χ*T* approaches the limit expected for 10 free Cr^3+^ ions with *g* = 2, χ*T*_300 K_ = 10*g*^2^*S*_i_ (*S*_i_ + 1)/8 = 18.7 emu·K/mol.

For decreasing temperature, the product χ*T* increases, reaching 32.7 emu K/mol at 1.8 K, (Figure 2b). This behavior points to an overall predominance of ferromagnetic interactions in the wheel. A similar χ*T*(*T*) curve was earlier found by Low et al.,^36^ although the reported value at 1.8 K was somewhat smaller (25 emu K/mol).

It is to be noted that the magnetic properties of the bulk (the saturation magnetization value, the initial *M*(*H*) slope, and χ*T* at a low *T*) changed when the sample was mechanically crushed into a powder or embedded in oil to fix the grains (see experiments in S1). We believe these changes may be explained by the severe dependence of the magnetic properties on even tiny distortions in the wheel Cr–O–Cr angles (vide infra). This may also be the reason for the different *M*(*H*) and χ*T*(*T*) data for powder {Cr_10_} samples reported by previous authors.^36,38^

### {Cr_10_} Anisotropy: Single-Crystal Results

To further investigate the magnetic anisotropy of {Cr_10_}, we performed magnetometry measurements on an oriented single crystal. The compound crystallizes in the triclinic *P*1̅ space group with *Z* = 1, thus all {Cr_10_} molecules are identically oriented in the crystal; the asymmetric unit contains five contiguous Cr^3+^ ions, and the other five ions in the ring are obtained by inversion. The molecule displays a close to D_5d_ symmetry (Figure 1).

Magnetization was measured as a function of the angle θ between the molecular fivefold quasi-symmetry axis and the direction of the applied magnetic field. The magnetization curves *M*(*H*) measured with the applied magnetic field *H* parallel (θ = 0°) and perpendicular (θ = 90°) to that quasi-fivefold ring axis clearly differ, evidencing a substantial anisotropy (Figure 2a). The measured *M*(*H*) curve with the field *H* at an intermediate angle falls in between, as may be expected. No saturation of the magnetization is observed up to 50 kOe (Figure 2a).

**Figure 3:**
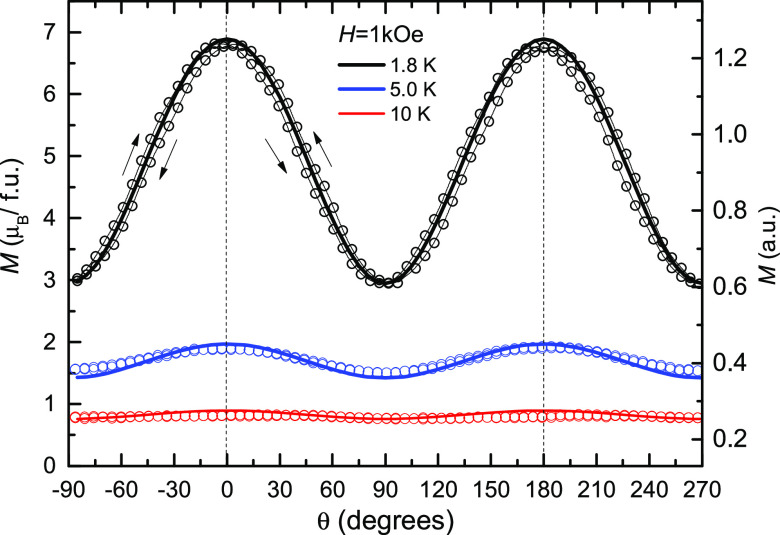
Single-crystal angular magnetization. Magnetization of the SC as a function of the angle between the easy axis of magnetization and the applied magnetic field (*H* = 1 kOe), measured at three different temperatures, *T* = 1.8, 5.0, and 10 K, together with calculated curves obtained using the Hamiltonian in eq 1 with *S* = 9, *g* = 2 and *D*/k_B_ = −0.045 K.

Figure 3 shows the magnetization of the SC measured as a function of the angle, *M*(θ), with a constant applied magnetic field *H* = 1 kOe, performed at three different temperatures, *T* = 1.8, 5, and 10 K. Though the sample is paramagnetic, an anisotropic magnetization response is clearly observed in the 1.8 K measurements. The maxima at θ = 0° and minima at θ = 90° identify the quasi-symmetry axis as an easy axis (EA) of magnetization, while a direction on the wheel’s mean plane is a hard axis (HA). The amplitude of the angle dependence decreases strongly with temperature and is practically negligible at 10 K.

To interpret quantitatively these results, we will assume that the strong exchange interaction approximation is applicable; therefore, the {Cr_10_} cluster may be described by a total cluster spin *S* in the giant-spin (GS) approximation.^39^ At a very low temperature, we will also assume that only the *S***=** 9 ground multiplet is thermally occupied. Thus, the cluster Hamiltonian with uniaxial anisotropy described by the zero-field splitting parameter *D*, under an applied external field at an angle θ with the anisotropy axis, is written as
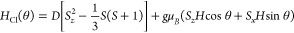
1The first term corresponds to the uniaxial anisotropy interaction, and the second to the Zeeman interaction with the applied magnetic field *H*. This Hamiltonian operates on the 9|,*s*_z_⟩ states. From the eigenvalues and eigenfunctions, the partition function was calculated, and the magnetization *M*(θ, *T*, *H*) was predicted, within the Boltzman statistics, at fixed temperature and field modulus, at varying θ. The parameters *D*/*k*_B_ = −0.045 K and *g* = 2 (isotropic) were used, the same previously found by EPR measurements.^37^ This kind of experiment and procedure was applied earlier to a single crystal of Mn_12_-PrCl.^40^ The calculated magnetization is shown in Figure 3 as solid curves for 1.8 K (black), 5 K (blue), and 10 K (red). The data are presented in arbitrary units because of several experimental indeterminations in the weighing of the very small sample. However, it must be noted that all of the data could be explained with the same scaling factor.

The χ*T*(*T*) curves for the SC with the applied field parallel and perpendicular to the easy axis of magnetization are shown in Figure 2b. We observe that while χ*T*(*T*) increases with decreasing the temperature down to 1.8 K when the field is applied with *H* || EA, the curve reaches a maximum and then decreases when *H* || HA.

So far, we can state that {Cr_10_} wheels at a low temperature show *S* = 9 as ground multiplet, uniaxial anisotropy (*D*/*k*_B_ = −0.045 K) with the EA perpendicular to the wheel plane.

### Heat Capacity

**Figure 4:**
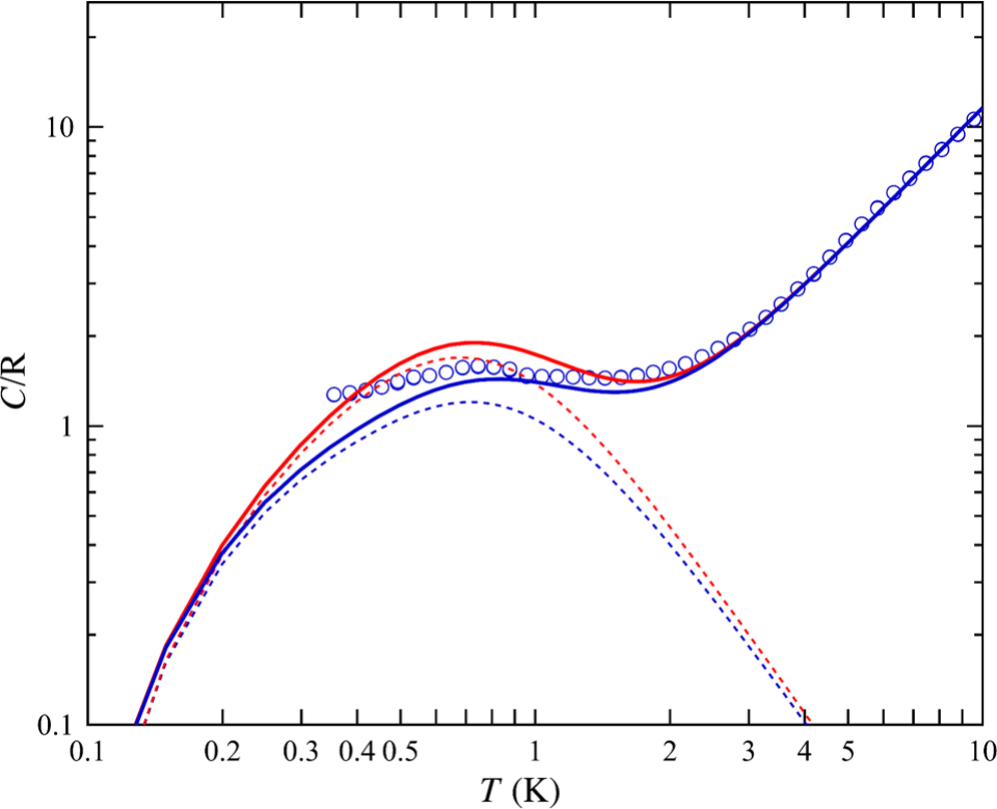
Heat capacity. Symbols: temperature dependence of the heat capacity of the powder sample measured at *H* = 0. Dotted lines: magnetic contribution *C*_m_(*T*) Schottky curve, calculated considering *D*/k_B_ = −0.045 K and only the *S* = 9 (blue), and the *S* = 9 ground state and excited *S* = 8 state with the same *D* values (red). Solid lines: total heat capacity, *C*(*T*) = *C*_m_(*T*) + *C*_L_(*T*), where the lattice contribution is *C*_L_(*T*) = *AT^n^*, with *A*/*R* = 0.35 K^–1.52^, *n* = 1.52.

The heat capacity as a function of the temperature in zero applied field was measured for the powder sample (Figure 4). The *C*(*T*) curve shows a typical Schottky shape, which is reasonably well fitted under the single-cluster Hamiltonian of eq 1, in agreement with the magnetometry results. By considering in the model also the excited *S* = 8 multiplet, as derived in the section from INS experiments (vide infra), split by the same anisotropy constant *D*/*k*_B_ = −0.045 K,^37^ the calculated curve (red line), lies slightly above the experimental data.

### XANES and XMCD at Cr K-Edge

**Figure 5:**
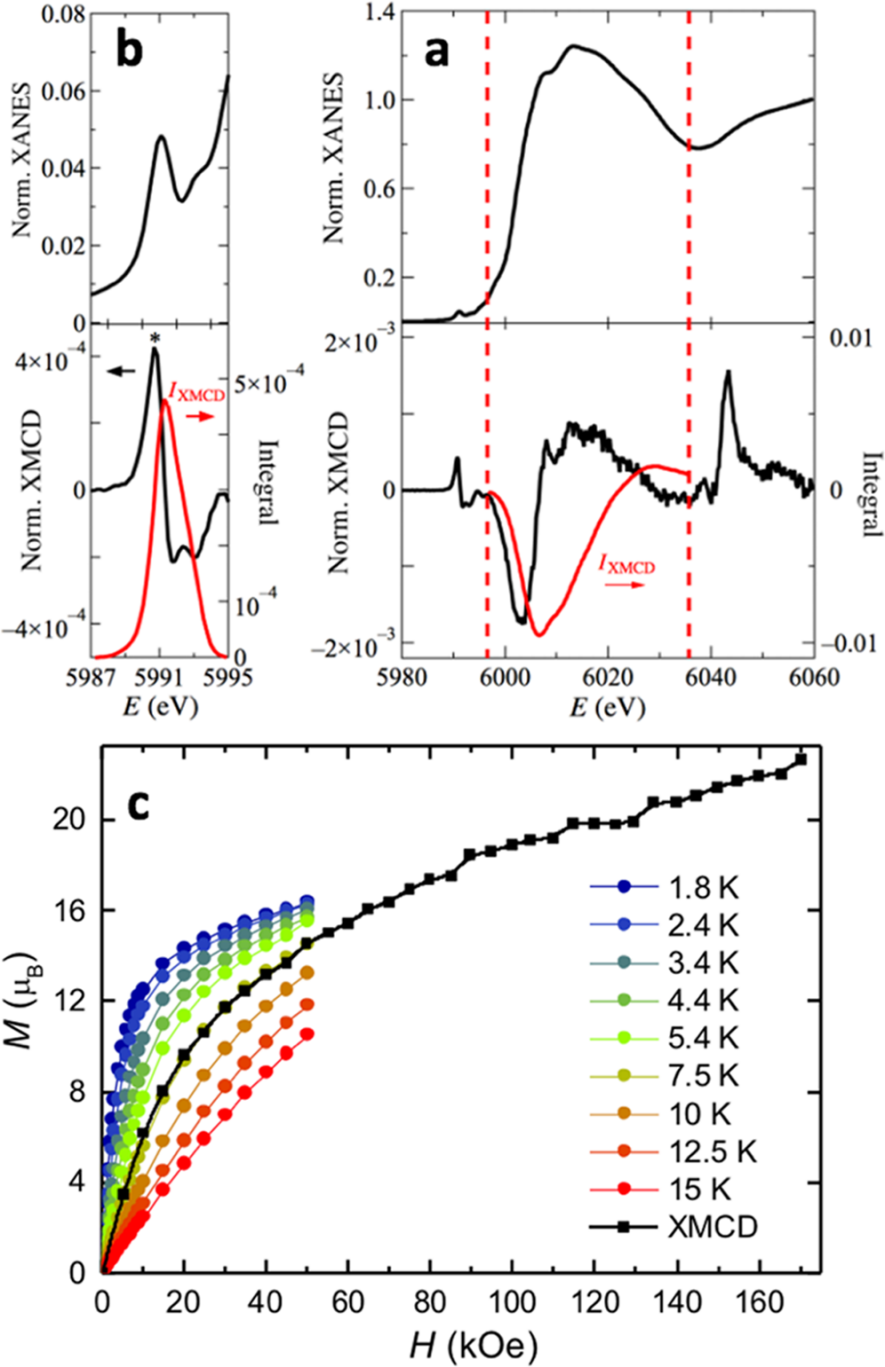
XANES and XMCD spectra at Cr K-edge. (a) Top: (•) normalized X-ray absorption spectroscopy (XANES), measured at 7.5 K and 170 kOe; bottom: (•) Normalized X-ray magnetic circular dichroism (XMCD); red, right scale: integrated XMCD, (*I*_XMCD_)_4p_. The vertical red dashed lines signal the lower and upper limits of integration; (b) zoom of the pre-K-edge region; bottom, red, right scale: integrated XMCD, (*I*_XMCD_)_pre-edge_; (c) XMCD(*H*) scaled to *M*(*H*) isotherms measured with SQUID magnetometry on the same sample. Note the matching of the XMCD(*H*) curve with *M*(*H*) at *T* = 7.5 K.

X-ray absorption spectroscopy (XANES) and X-ray magnetic circular dichroism (XMCD) experiments at the Cr K-edge were performed on a powder sample. Figure 5a shows the XANES and XMCD spectra measured at 170 kOe and 7.5 K. The photon energy range spans 5980 eV < *E* < 6060 eV. Three regions (separated by vertical dashed lines in Figure 5a) should be distinguished.

The pre-edge region, below *E* ≈ 5997 eV involves transitions from 1s to unoccupied 3d states, which may be purely quadrupolar or dipolar if the final states are symmetry-allowed hybridized 3d–4p states. Above *E* ≈ 5997 eV, XANES and XMCD are dominated by transitions from the 1s to the 4p empty states. Finally, at energies above 6035 eV, super-Coster–Kronig multielectron excitations take place, involving two-electron transitions from 1s to 4p and from shallow core 3p to 3d states.^41,42^ In this third region, these multielectron excitations dominate the XMCD spectrum.

The XANES and XMCD spectra at the pre-edge energy region are shown in expanded view in Figure 5b.

Two peaks are clearly observed in XANES, with corresponding maxima and two minima in the XMCD spectrum. The feature at the lowest energy (5990.85 eV) may be ascribed to the 1s → t_2g_ transition.^43^ A direct comparison with the spectra obtained in reference compounds for Cr oxidation states ions^41,44,45^ leads us to conclude that chromium ions in {Cr_10_} are in trivalent state, Cr(III).

Since the initial state in the K-edge, 1s, is not spin–orbit split, XMCD is originated only in the orbital imbalance of the final states. As shown by Thole and Carra,^46,47^ the integrated XMCD signal, *I*_XMCD_, is related through the orbital sum rule to the ground-state expectation value of the orbital moment ⟨*L*_z_⟩ of the final state, which may be 4p for dipolar transitions or 3d for quadrupolar ones.^48^ As shown in Figure 5b, the integral of the pre-edge XMCD, (*I*_XMCD_)_pre-edge_, is very small compared to (*I*_XANES_)_pre-edge_ (Figure S2.1), therefore indicating that ⟨*L*_z_⟩_3d_ is negligible. Likewise, in Figure 5a, we show that (*I*_XMCD_)_4p_ = 9 × 10^–4^ eV is also very small, thus, ⟨*L*_z_⟩_4p_ is negligible. As we find that both orbital contributions from 3d and 4p electrons are negligible, the Cr magnetic moment is totally of spin character. Therefore, an isotropic *g* factor is fully justified to describe the Zeeman effect.

Besides, it is known that in compounds comprising just one magnetic element, the XMCD at the K-edge is proportional to the magnetization in an applied field, and therefore, to the magnetic moment of the absorbing atom.^41^ By scaling the experimental XMCD K-edge spectra to that of a reference Cr(III) compound, namely, *trans*-[Cr(III)Cl_2_(pyridine)_4_](ClO_4_)·1/4H_2_O, in short {Cr(III)}, an estimation of the average Cr moment in the field direction may be extracted. The magnetic moment of {Cr(III)} is saturated at 3 K and 170 kOe, and amounts to 3.1 μ_B._^41^ This value needs to be multiplied by a factor of 0.68 to scale with that of {Cr_10_} (see Figure S2.2), yielding a value of *m*_Cr_ = 2.1 μ_B_ per Cr ion, at *T* = 7.5 K and 170 kOe for {Cr_10_}. Since the coordination and distances around the Cr^3+^ ion are different in {Cr(III)} and {Cr_10_}, the spectral shapes are quite different. However, we consider this estimation correct, as we show below.

The isothermal, field-dependent XMCD signal was measured by changing the helicity of the beam at a fixed photon energy of 5990.85 eV (* in Figure 5b). This incident energy corresponds to the Cr pre-edge, whose XMCD peak is sensitive to the 3d magnetic moment. When the field is varied in the range 0 < *H* < 170 kOe, at fixed temperature *T* = 7.5 K, the XMCD(*H, E*_*photon*_ = 5990.85 eV) curve is proportional to *M*(*H*), i.e the magnetic moment from the 3d states, which is dominant with respect to any other contribution. In Figure 5c, the normalized XMCD(*H*) per Cr ion is shown in the right axis compared with the *M*(*H*) isotherms measured with SQUID magnetometry on the same powder sample. The XMCD(*H*) agrees perfectly with the isotherm for *T* = 7.5 K. The magnetization deduced at 170 kOe is *M*(*H*) = 22.6 μ_B_, which corresponds to a value of *m*_Cr_ = 2.26 μ_B_, per Cr ion which is very close to the estimation derived from the scaling of the XMCD spectrum described in the previous paragraph.

It is noteworthy that the XMCD(*H*) curve continuously increases its value over the *M*_s_ = 18 μ_B_ saturation value expected for *S* = 9, implying that the 10 spins of the molecule are progressively oriented by the increasing field. For even higher fields, one may expect that the limit for *S* = 15 i.e. *M*_s_ ≈ 30 μ_B_ may be reached, once all of the individual spins are maximally oriented along the field direction.

### *Ab Initio* and DFT Calculations

**Figure 6:**
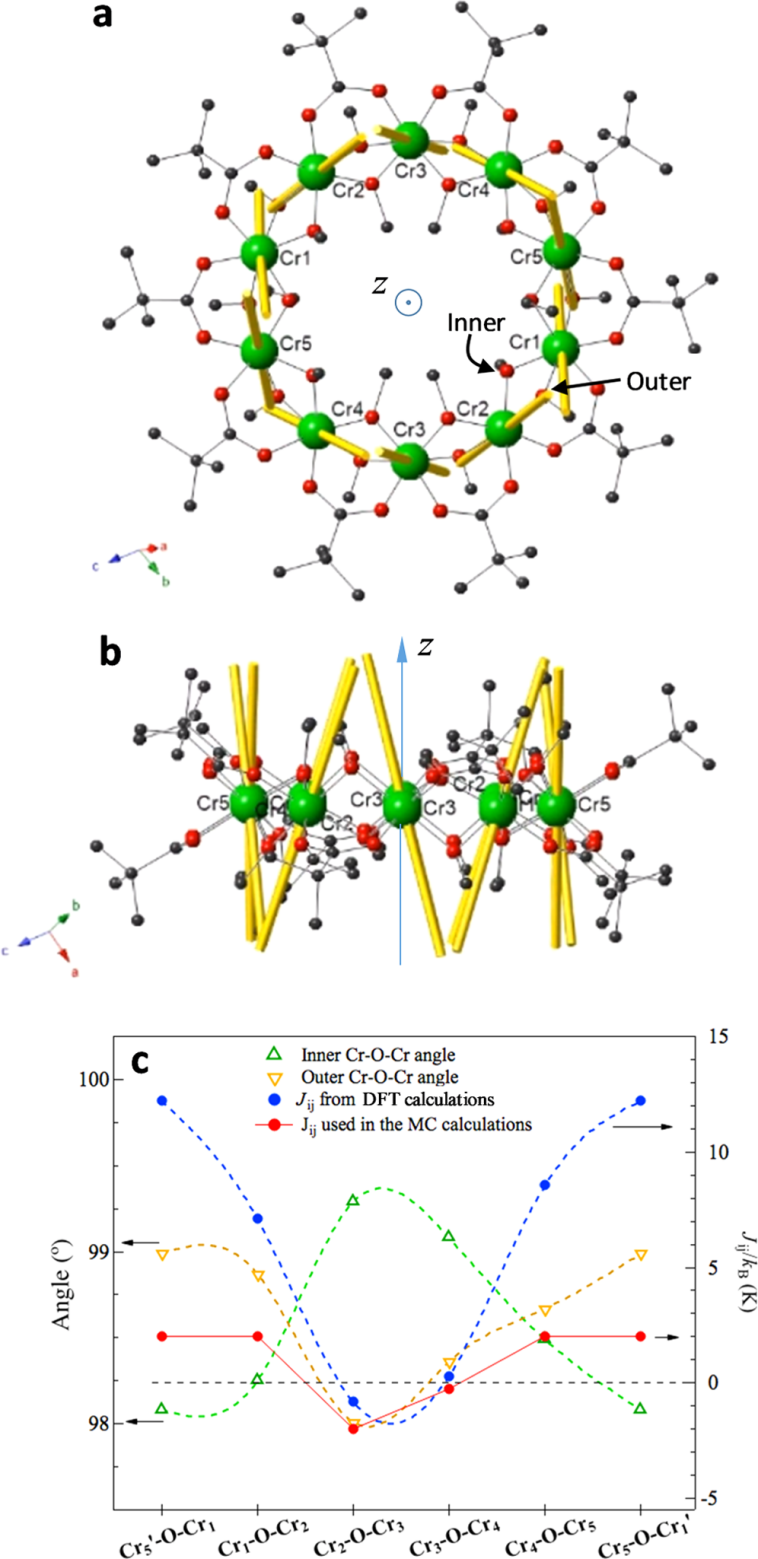
(a) Easy axes of magnetization (EAM) calculated by *ab initio* for the {Cr_10_} ring. The inner and outer Cr–O–Cr alkoxide bridges, the carboxylate bridges, and Cr ions numbering is that of the asymmetric unit as in Low et al.;^36^ (b) lateral view; and (c) correlation between the angle Cr–O–Cr alkoxy bridges and DFT-calculated *J*_ij_ coupling constants between spins of ions Cr_i_ and Cr_j_ for each of the five Cr^3+^ ions in the asymmetric cell of the {Cr_10_} wheel.

The multispin (MS) cluster Hamiltonian approximation consists of two terms, the first encompassing zero-field splitting by ligand field interactions on the single ions, *H*_a_, and the second comprising the interion interaction, either dipolar or exchange, *H*_ex_
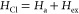
2Here, *H*_a_ = ∑_*i*=1_^10^*H*_*i*_ is a sum over the 10 Cr^3+^ spins, where *H*_*i*_ = *D*_*i*_*S*_*iz*′_^2^ + *E*_*i*_(*S*_*ix*′_^2^ – *S*_*iy*′_^2^) is the second-order single-ion zero-field splitting Hamiltonian of each Cr_i_ with axial and rhombic terms. We performed *ab initio* calculations to determine the local anisotropy of the Cr^3+^ ions in the {Cr_10_} ring. The axial (*D*_i_) and rhombic (*E*_i_) local anisotropy terms were calculated for each of the five inequivalent Cr^3+^ ions of the asymmetric unit cell (numbered Cr1 to Cr5 as in ref (36)) using the package ORCA (see S3). The values of *D*_i_/*k*_B_ ≈ −0.25 K and *E*_i_/*D*_i_ of all ions were found to be similar, with a slight depletion around ion Cr3 (see Table 1). The anisotropy is axial (*D*_i_ < 0) for all five ions with an important rhombic contribution (*E*_i_/*D*_i_ in the range 0.25–0.33). In all cases, there is a local easy anisotropy axis close to the {Cr_10_} ring’s mean axis (Figure 6a,b).

**Table 1:** Anisotropy Constants^a^

Cr ion	*D*_i_/*k*_B_ (K)	*E*_i_/*D*_i_
Cr1	–0.32	0.33
Cr2	–0.25	0.28
Cr3	–0.20	0.25
Cr4	–0.27	0.26
Cr5	–0.33	0.33
{Cr_10_} cluster	*D*/*k*_B_ (K)	*E*/*D*
EPR^37,38^	–0.045(4)	
INS	–0.045(2)	
calculated	*E*_a_/*D*_a_	*E*_a_/*D*_a_
	–0.033	0.15

a *Ab initio* calculated anisotropy constants *D*_i_ and *E*_i_/*D*_i_ ratio for the five Cr^3+^ ions in the asymmetric unit cell. Also, calculated and experimental cluster anisotropy *D* and *E*/*D* ratio.

The interion, intracluster exchange interaction may be expressed in terms of the single-ion spins, *S*_i_, with a Heisenberg–Dirac–van Vleck Hamiltonian, neglecting anisotropic exchange, dipolar and intercluster interactions
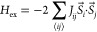
3

with ⟨*ij*⟩ denoting all interactions between near-neighbor (n.n.) Cr^3+^ ions with spins *S*_i_ = 3/2. The constants *J*_ij_ were calculated by DFT in ORCA using B3LYP hybrid functional for each of the five Cr–Cr couples in the asymmetric cell. The theoretical results, summarized in Figure 6c and Table S4, evidence a clear asymmetry in the interaction around the Cr3 ion: indeed, while *J*_23_ is AF and *J*_34_ only slightly FM, all other interactions are FM (see Figure 6c).

The calculated decreased values of *D*_i_ and *J*_ij_ around Cr3 can be correlated with small variations in the angles of the Cr-O-Cr alkoxide bridges connecting this ion to its n.n. It is noted that there are two types of Cr–O–Cr alkoxide bridges (see S4 and Figure 6a). The *outer* one points upward (alternatively downward in the next Cr–O–Cr bridge) with respect to the {Cr_10_} mean plane with the dihedral angle between the Cr–O–Cr and {Cr_10_} planes all in the range 105–108°. In contrast, the *inner* bridge points toward the molecule plane’s normal axis and downward (alternatively upward) with the Cr–O–Cr plane angle of ≈45° with respect to the {Cr_10_} plane. Notably, as shown in Figure 6a,b, both Cr–O–Cr bridges for Cr3 are peculiar: it is the only case in this molecule where the Cr–O–Cr angle of the inner bridge is larger than that of the outer one, on both sides of the ion, and this small angular difference drastically reduces the coupling constant. Although the absolute values for the interactions might somehow depend on the chosen functional, the results undoubtedly demonstrate the existence of a rupture in the symmetry of the interactions associated with small structural changes.

The use of heteroleptic carboxylate/alkoxide bridging ligands in {Cr_10_} favors the formation of ferromagnetic interactions between the Cr^3+^ ions, likely due to the orbital counter-complementarity effect introduced by the carboxylates.^50,51^ However, we observe that the magnitude and sign of the exchange constant finely depend on structural parameters, in agreement with results of magneto-structural DFT studies performed on particular Cr_2_ dimeric model systems.^51^

To promote SMM behavior, it is not enough to have a large spin *S* and single ions with large anisotropy, but parallel alignment between the anisotropy axes of the ions is also important.^51^Figure 6a,b shows the different deviation of the local anisotropic axes of each Cr_i_ with respect to the wheel’s *z*-axis. The deviation angle and coupling *J*_ij_ are correlated, as shown in Figure S4.1, which stems from the fact that both magnitudes depend on the local structural details around each Cr_i_. The minimum deviation angle (∼17°) and largest AF exchange correspond to Cr3, for which the difference between the Cr–O–Cr angle of the inner and outer bridge is maximum and positive, whereas the largest deviation (26°) corresponds to Cr1, with the largest FM and maximum negative difference between the angles of the inner and outer Cr–O–Cr bridges.

A different approach to describe the cluster Hamiltonian is to apply the *giant-spin* (GS) approximation, as has been used already in eq 1, *H*_Cl_ = *DS*_*z*_^2^ + *E*(*S*_*x*_^2^ – *S*_*y*_^2^), where *S* is the total cluster spin, and *x*, *y*, and *z* are the cluster axes, with *z* perpendicular to the wheel plane. The isotropic exchange interaction between Cr spins is already implicitly considered when the ground state with total spin *S* is assumed; therefore, the *J*_ij_ parameters do not appear explicitly in this formulation. The next step is to correlate the multispin Hamiltonian description with the giant-spin one.

The transformation from the local anisotropy of Cr^3+^ ions, *D*_i_, and interion interaction anisotropy to an equivalent effective anisotropy of the {Cr_10_} wheel, *D*, can be carried out in the strong isotropic exchange limit, and assuming collinearity of the spins, by means of a linear combination of the former, with *d*_i_ and *d*_ij_ coefficients, which may be calculated taking into account symmetry relations. Then, according to ref (52), the following relations are fulfilled
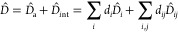
4
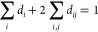
5where *D̂*_*i*_ are local anisotropy tensors, the *D̂*_*ij*_ tensors contain interion anisotropy terms as, e.g., dipolar and exchange interactions, and *D̂* is the anisotropy tensor of the full molecule. This method has been successful in several analyses of anisotropy in dinuclear clusters.^53−55^

The coefficients *d*_*i*_ have been calculated following the method described in ref (52) and are collected in Table S3.3. Once the *d*_i_ values are calculated, we proceed to calculate the *D̂*_a_ = ∑_*i*_*d*_*i*_*D̂*_*i*_ tensor. The *D̂*_*i*_ tensors must be written in a common coordinate system, in fact, that of the Cr3 (Figure S3.1). It gives rise to a nondiagonal molecular tensor which has to be diagonalized to obtain the equivalent anisotropy constants *D*_a_ and *E*_a_, from single-ion anisotropies, i.e., excluding interion interactions. The result is *D*_a_/*k*_B_ = −0.033 K and *E*_a_/*k*_B_ = −0.0048 K (*E*_a_/*D*_a_ = 0.0145). Hence, the magnetic anisotropy of the {Cr_10_} ring is uniaxial with a rhombic distortion smaller than that of the constituent Cr^3+^ ions, and the anisotropy direction (*z*′) is very close to the perpendicular to the wheel’s mean plane (≈2.7° out of the molecule’s *z*-axis).

The difference between the experimental value *D*/*k*_B_ = −0.045(2) K and the calculated value *D*_a_/*k*_B_ = −0.033 K may be ascribed to the uncertainty originated by the approximations applied in the *ab initio* methods, or in the deviation from collinearity, but it could also have a contribution from the interion interaction, *D*_int_ term in eq 4. Considering *D*_int_/*k*_B_ = −0.012 K as an upper threshold for the interaction contribution, an estimate of the maximum Cr–Cr interion exchange anisotropy can be made using eq 4 and the calculated average coefficient *d*_CrCr_ = 0.0428 (S5), yielding a negative value *D*_CrCr_/*k*_B_ = −0.028 K. Hence, its effect is to reinforce the local uniaxial anisotropy, perpendicular to the wheel’s plane.

All in all, these calculations allow us to conclude that the full {Cr_10_} ring behaves as a magnetic unit with axial anisotropy of lower rhombicity than the constituent ions, in agreement with the interpretation of EPR results,^37^ which yielded the experimental value *D*/*k*_B_ = −0.045 K (Table 1), and the *M*(θ) results obtained in this paper.

### Monte Carlo Calculations

The thermomagnetic properties can be calculated, within the Boltzman equilibrium statistics, using the cluster MS Hamiltonian in terms of the single-ion spins *S*_i_
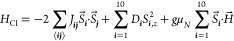
6where the first term describes the exchange interaction coupling, with ⟨***ij***⟩ denoting all interactions between the n.n. *S*_i_ = 3/2 spins in the {Cr_10_} ring; spins are numbered from 1 to 10 following the numbering of the ions in asymmetric unit from 1 to 5, and 6 to 10 for the ions obtained by inversion (Cr1 → Cr6, etc.). The second term accounts for the zero-field splitting produced by the single-ion anisotropy of each Cr^3+^ site (*D*_i_), and the last term is the Zeeman splitting with *g* = 2. Since the full diagonalization of that Hamiltonian requires to work in a functions space of dimension (2 × 3/2 +1)^10^, an alternative approach is to use Monte Carlo (MC) simulations. Classical MC methods have proved useful to rationalize the magnetic properties of 3d clusters with a large number of ions.^59,60^

**Figure 7:**
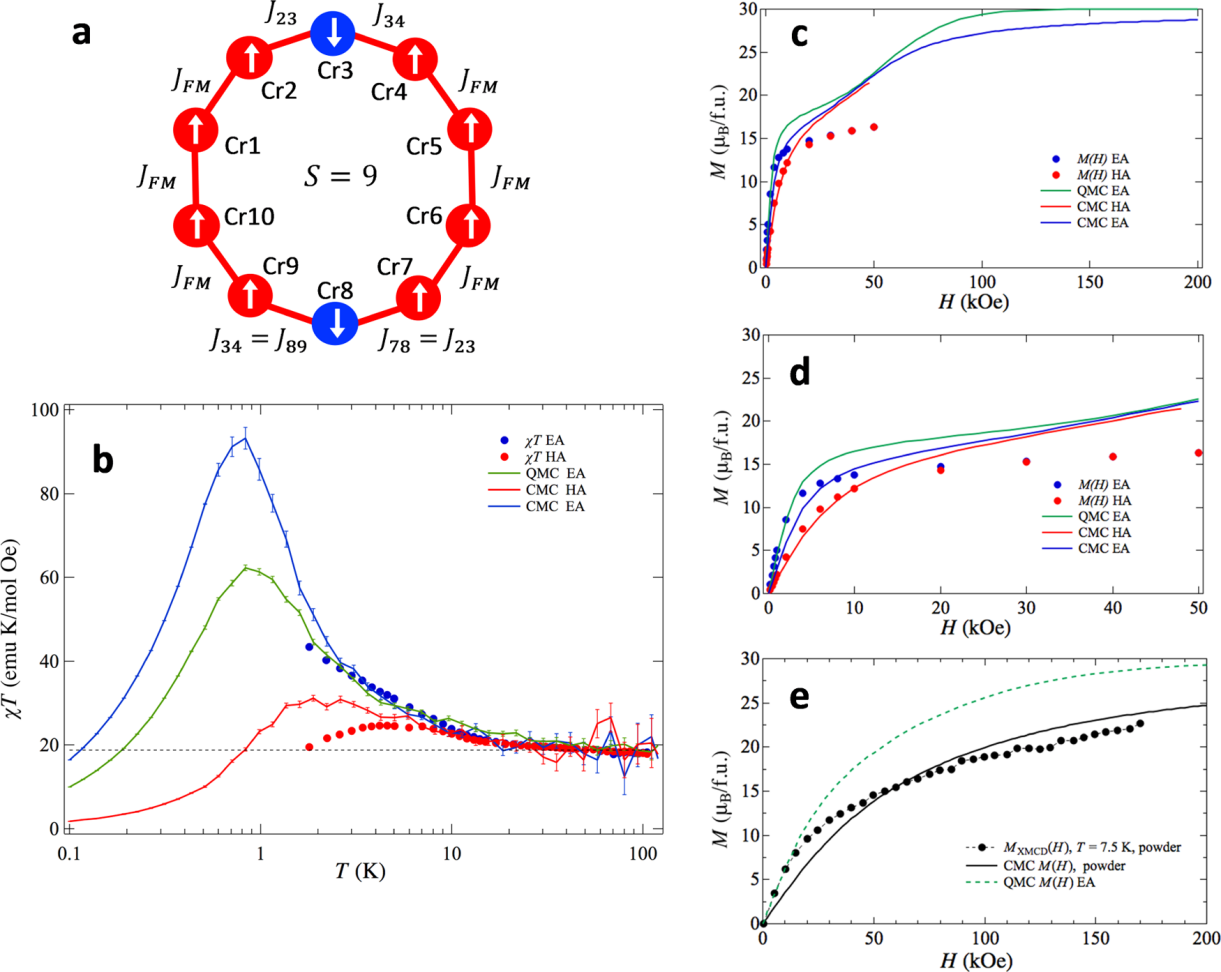
{Cr_10_} magnetic modeling. (a) {Cr_10_} coupling scheme and (b–e) magnetic curves calculated with a classical Monte Carlo model along the EA (blue line) and HA (red lines) directions and quantum Monte Carlo only for the EA direction (green lines), with the parameters set *J*_FM_/k_B_ = 2.0 K, *J*_23_/*k*_B_ = −2.0 K, *J*_34_/*k*_B_ = −0.25 K, *D*_i_/*k*_B_ = −0.31 K. Experimental data with the applied field in the EA (blue full circles) and HA (red full circles) directions. (b) Magnetic susceptibility χ*T*(*T*) at 1 kOe; (c) magnetization *M*(*H*) at *T* = 1.8 K; (d) zoom-in of the *M*(*H*) up to 50 kOe; and (e) calculated classical MC and XMCD experimental results of *M*(*H*) for the powder sample at 7.5 K. The quantum MC simulation for the EA direction is also included (green dashed line).

We used the MC method as implemented in ALPS^56,57^ for ten 3/2 spins in a ring and a distribution of exchange constants *J*_ij_ as shown in Figure 7a, where the {Cr_10_} wheel is divided into two sets of five Cr^3+^ ions as in the crystallographic asymmetric unit. The results of the DFT calculations were used as a trend and simplified as follows: the n.n. interactions were taken as identical and ferromagnetic (*J*_FM_), except around two Cr^3+^ ions on opposite sides of the wheel, denoted Cr3 and Cr8 as in the previous section (Figure 7a), which were taken as antiferromagnetic and asymmetric (*J*_23_ = *J*_78_ ≠ *J*_34_ = *J*_89_). This ensures a ground state with total spin *S* = 9 in zero magnetic field. For the MC simulations, the cluster anisotropy value obtained by EPR *D*/*k*_B_ = −0.045 K, normal to the {Cr_10_} wheel plane and confirmed by INS (see next section), was translated into an average single-ion anisotropy *D*_i_/*k*_B_  = (*D*/*k*_B_)/∑_*i*_*d*_*i*_ = −0.31 K using the strong exchange limit approximation^58^ with neglected interion anisotropic contributions; this single-ion anisotropy *D*_*i*_ was set equal for all ions and no rhombic anisotropy constant was included. The classical MC method was used to derive the magnetization along the easy anisotropy axis (EA) and perpendicular to that direction (HA). The susceptibility as a function of temperature χ(*T*) was calculated as *M*/*H* for an external applied field of 1 kOe, as it was carried out experimentally.

Additionally, quantum MC simulations, using the directed loop code in the stochastic series expansion representation,^61^ were performed only for the component parallel to the anisotropy axis, since for the in-plane component, a sign problem^56,57^ is produced.

The field dependence of the magnetization at 1.8 K and the temperature dependence of the susceptibility of this {Cr_10_} model were calculated searching for a set of exchange coupling constants consistent with the experimental data. Figure 7b–e shows the results for the coupling constants *J*_FM_/*k*_B_ = 2.0 K, *J*_23_/*k*_B_ = −2.0 K, and *J*_34_/*k*_B_ = −0.25 K. The anisotropy in the magnetic susceptibility as a function of temperature is explained qualitatively for both the EA and HA directions (Figure 7b). The anisotropy in the magnetization measured for the SC at 1.8 K (Figure 7c,d) is also explained qualitatively, although the predicted curve quickly diverges from the experimental data for larger fields. Besides, the MC simulation approaches the XMCD(*H*) curve measured for the powder at 7.5 K in the same range of fields (Figure 7e). For low magnetic field values, the classical MC simulation for the present magnetic system yields similar values to those of the quantum MC down to ≈2 K, while as the field increases the two models differ.

Our model predicts that the *M*(*H*) curve increases continuously over the maximum value expected for an *S* = 9 total spin (18 μ_B_), linearly approaching the 30 μ_B_ limit for the totally polarized spin state. Thus, the *S* = 9 ground state holds for a limited range of magnetic field, about *H* = 100 kOe. For higher applied fields, magnetic states with a larger *S* and *S*_z_ become the ground state. The saturation value of 30 μ_B_, expected for the totally polarized set of ten 3/2 spins (*S* = 15), has not been reached in the present work up to 170 kOe.

The present coupling scheme is supported by the INS measurements (*vide infra*). We note that other sets of parameters (*J*_ij_, *D*_*i*_,) are able to fit the magnetic data. Indeed, the experimental magnetization slowly tends toward the maximum value for a ten 3/2 spins system as the magnetic field increases, and the faster drop of the HA component of χ*T* at low temperatures suggest that the actual distribution of exchange interactions within the {Cr_10_} ring includes a higher antiferromagnetic character. However, as we will show in the next section, this is incompatible with the INS results.

### Inelastic Neutron Scattering

Although the results presented above have clearly confirmed an *S* = 9 ground state and a wheel’s uniaxial magnetic anisotropy *D*/*k*_B_ = −0.045 K, which is proved to be along the symmetry axis of the {Cr_10_} molecule, we still lack conclusive information about the excited states. Inelastic neutron scattering (INS) is a powerful spectroscopy technique to determine the magnetic exchange splitting and reveal the energy of the excited states directly. This technique has been applied very successfully in the study of magnetic clusters (e.g., on Fe_8_,^62^ Mn_12_^63^ and others^64^) and rings (e.g., {Cr_8_},^19,20^ {Cr_7_M},^27,28,31^ {Cr_8_Cd}^32^).

In the INS experiment, the incoming neutron exchanges energy and momentum with the sample and the energy and momentum of the outgoing neutron are analyzed. The states of the sample are therefore determined by the difference ℏω between the incoming and outgoing neutron energies.

**Figure 8:**
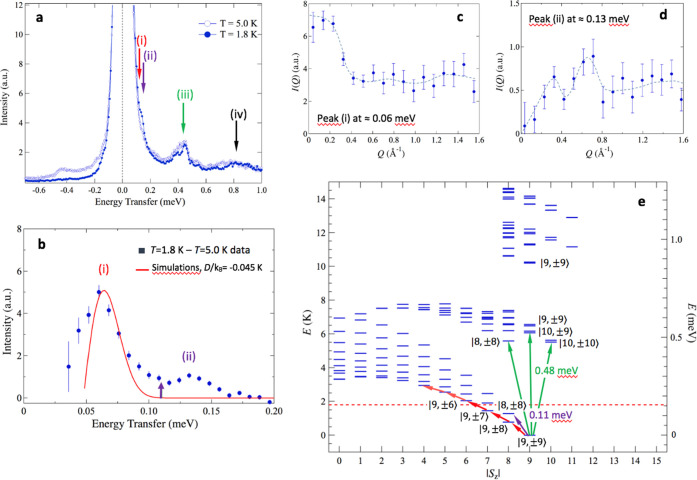
Inelastic neutron scattering (INS) spectra of {Cr_10_}. (a) Spectra measured at *T* = 1.8 and 5.0 K on the LET spectrometer, with *E*_i_ = 1.5 meV.(b) Low-energy loss spectrum obtained from the difference between the *T* = 1.8 and 5.0 K spectra shown in (a). The low-energy peak is compared to the simulation of the difference spectrum (red full line) for the same two temperatures for only intramultiplet *S* = 9 transitions with *D*/*k*_B_ = −0.045 K, *E*/*D* = 0. The purple arrow indicates the energy of the calculated |9, ± 9⟩ to |8, ± 8⟩ transition as shown in (e); (c) integral intensity, *I*(*Q*), of the experimental peak at ℏω≈ 0.06 meV and (d) *I*(*Q*) for the peak at ≈ 0.13 meV. In (c) and (d), dashed lines are a guide to the eye. (e) Calculated energy levels as a function of *S*_z_ calculated by diagonalization of the exchange Hamiltonian in eq 6 for the coupling scheme shown in Figure 7a. For *S*_z_ < 8, only the first 10 levels calculated with the Lanczos method are shown, while for *S*_z_ ≥ 8, all of the levels up to 1.3 meV are depicted. Red arrows: excitations within the *S* = 9 multiplet, fulfilling the selection rule Δ*S* = 0, Δ*S*_z_ = ±1. Purple and green arrows: excitations complying with the selection rule Δ*S* = 1, Δ*S*_z_ = 0, ±1. The horizontal dotted line shows the energy corresponding to the measurement temperature.

The energy spectrum *S* (ℏω) is obtained by integrating the scattered intensity *S*(*Q*, ℏω) over the scattered intensity in the experimentally accessible Q-space (0 < *Q* < 1.6 for *E*_i_ = 1.5 meV); see Figure 8a. The *T* = 1.8 K spectrum displays visible peaks in the neutron energy loss region, i.e., the sample’s excitations (ℏω > 0), at the energy transfers of 0.4 meV (peak labeled as (iii) in the figure) and 0.8 meV (peak (iv)), and a small but evident feature at ∼ 0.15 meV (peaks (i)–(ii) on the right-hand side of the elastic peak). In the neutron energy gain region (ℏω < 0), these peaks are not discernible, except for the small shoulder of the elastic peak. This reflects transitions from the {Cr_10_} wheel’s magnetic ground and low-energy states or lattice excitations. By the temperature and Q-dependence of the observed excitations, we can deduce that they are predominantly of magnetic origin. On the contrary, at *T* = 5 K, higher-energy peaks become more unstructured in the ℏω > 0 region, while they are now visible in the ℏω < 0 region, and the intensity of the shoulder on the elastic peak shifts from the neutron energy loss to the energy gain region. This is consistent with the population of excited states at 5 K. To extract this low-energy peak from the elastic contribution, we subtract the 5 K spectrum from the *T* = 1.8 K one. The difference between both spectra in the range 0 < ℏω < 0.6 meV, is shown in Figure 8b, which evidences that the initial shoulder of the elastic line actually comprises two well-resolved peaks ((i) and (ii)).

To understand which type of magnetic states are involved in these two intensity peaks, we extracted the neutron scattering intensity as a function of the momentum transfer, *I*(*Q*), for both excitations. The *I*(*Q*) can help to understand what type of magnetic transitions occur.^64^ The intensity of the peak at 0.06 meV (i) tends to a nonzero constant value for *Q* → 0 (Figure 8c), as expected for intramultiplet transitions in neutron spectra, *i.e*., with no change in the total spin (Δ*S* = 0), while for the peak at 0.13 meV (ii) that integral intensity drops to zero for *Q* → 0 (Figure 8d), as predicted for magnetic transitions between multiplets of different total spin (Δ*S* = ± 1). Therefore, these peaks are associated with transitions between states of the *S* = 9 ground state multiplet split by magnetic anisotropy, and transitions to excited states belonging to multiplets with either *S* = 8 or 10.

Further insight into the magnetic transitions displayed by the neutron spectrum can be gained by calculating the energy levels, using the same Hamiltonian of eq 6 and the intracluster interactions scheme and constants as in the Monte Carlo simulations above with no applied magnetic field or dipolar interactions. However, unlike the classical Monte Carlo simulation, which only provides the ground state properties, now we need a quantum calculation to get the excited states. Although this implies to diagonalize a matrix of dimension 4^10^, it can be factorized in smaller matrices of fixed *S*_z_. It should be noticed that when the axial anisotropy is included, the total spin *S* is not a good quantum number anymore, but *S*_*z*_ still is; however, we will still use states labeling as |α, *S*, *S*_*z*_⟩ as an approximation (α denotes different single-ion spin configurations producing total spin third component *S*_*z*_), which allows us to track the states from the isotropic case, where *S* is a good quantum number (see Figure S6). For *S*_*z*_ ≥ 8, the dimensions of the matrices to be diagonalized were small enough to render practical a standard full diagonalization method, while for total spin values *S*_*z*_ < 8, we chose a Lanczos sparse diagonalization method to obtain the lowest energy levels.

Figure 8e shows the obtained energy levels as a function of *S*_z_ for the coupling scheme and *J*_ij_ values obtained in the Monte Carlo simulations above. Since in the 1.8 K spectrum we are observing transitions from the lowest *S* = 9 total spin states, states in the multiplets of total spin *S* ≥ 7 or *S* ≥ 11 are not detected because of the neutron scattering selection rules (see below).

To simulate the lowest-energy peak in the spectrum, we used a simpler approach starting from the energy-level scheme of the particular case *D*_i_ = 0, and then we added the cluster’s uniaxial magnetic anisotropy in the strong exchange limit approximation as a perturbation acting in an *S* fixed space. That is, we take the results from the MS approximation, as starting values in the GS approximation. Then, at *H* = 0
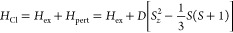
7

The anisotropy term produces spliting of the *S* = 9 and *S* = 8 multiplets. The splitting on these two multiplets may be regarded with some caution since there may be some mixing between states neglected by the model.^65^ The neutron scattering function for magnetic INS transitions within the ground *S* = 9 state (in the strong exchange approximation, GS) can be written as^63^
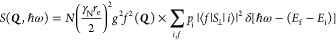
8

with the matrix elements between initial |*i*⟩ and final |*f*⟩ states in the |*S,S*_*z*_⟩ spin states base, S_⊥_ is the spin component perpendicular to the momentum transfer vector ***Q***, and
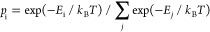
9

is the thermal population for the state. For a polycrystalline sample
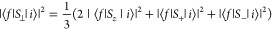
10

In the experimental spectra, the delta functions are convoluted with the experimental resolution Gaussian function^66^ of 21 μeV width in the present case.

First, we simulated the INS spectrum for excitations with Δ*S* = 0, Δ*S*_z_ = ±1, which are depicted by the red arrows in Figure 8e. The predicted INS spectra for 1.8 and 5 K have been substracted (red line in Figure 8b) and compared to the difference of the experimental spectra at the same temperatures. The predicted curve for *S* = 9, *D*/*k*_B_ = −0.045(2) agrees with the experimental peak (i) centered at 0.06 meV.

Second, the lower-energy excitations complying with the Δ*S* = 1, Δ*S*_z_ = 0, ± 1 selection rule are depicted in Figure 8e by purple arrows, which predict transitions dominated by the |9, ± 9⟩ → |8, ±8⟩ excitations of 0.11 meV, in good agreement with the peak centered at (ii) 0.13 meV observed experimentally.

In addition, the model is also able to account for the features in the neutron spectrum at larger energy transfer values. The doublet observed at (iii) 0.4 meV can be assigned, respectively, to intermultiplet transitions from *S* = 9 to *S* = 8 and *S* = 10 multiplets, as shown in Figure 8e (green lines). The high-energy feature at (iv) 0.8 meV can be explained as caused by transitions to higher multiples of the *S* = 8, 9, and 10, in agreement with the previously suggested estimation of ≈ 10 K (≈ 0.86 meV) from EPR measurements.^37^

All in all, the agreement of the predictions with the experimental peaks corresponding to the Δ*S* = 0 and Δ*S* = ±1 transitions supports our microscopic model comprising an *S* = 9 ground state and a close next excited *S* = 8 split by an anisotropy constant *D*/*k*_B_= −0.045 K, while the *S* = 10 multiplet lies ≈0.5 meV above.

### Dynamic Relaxation

**Figure 9:**
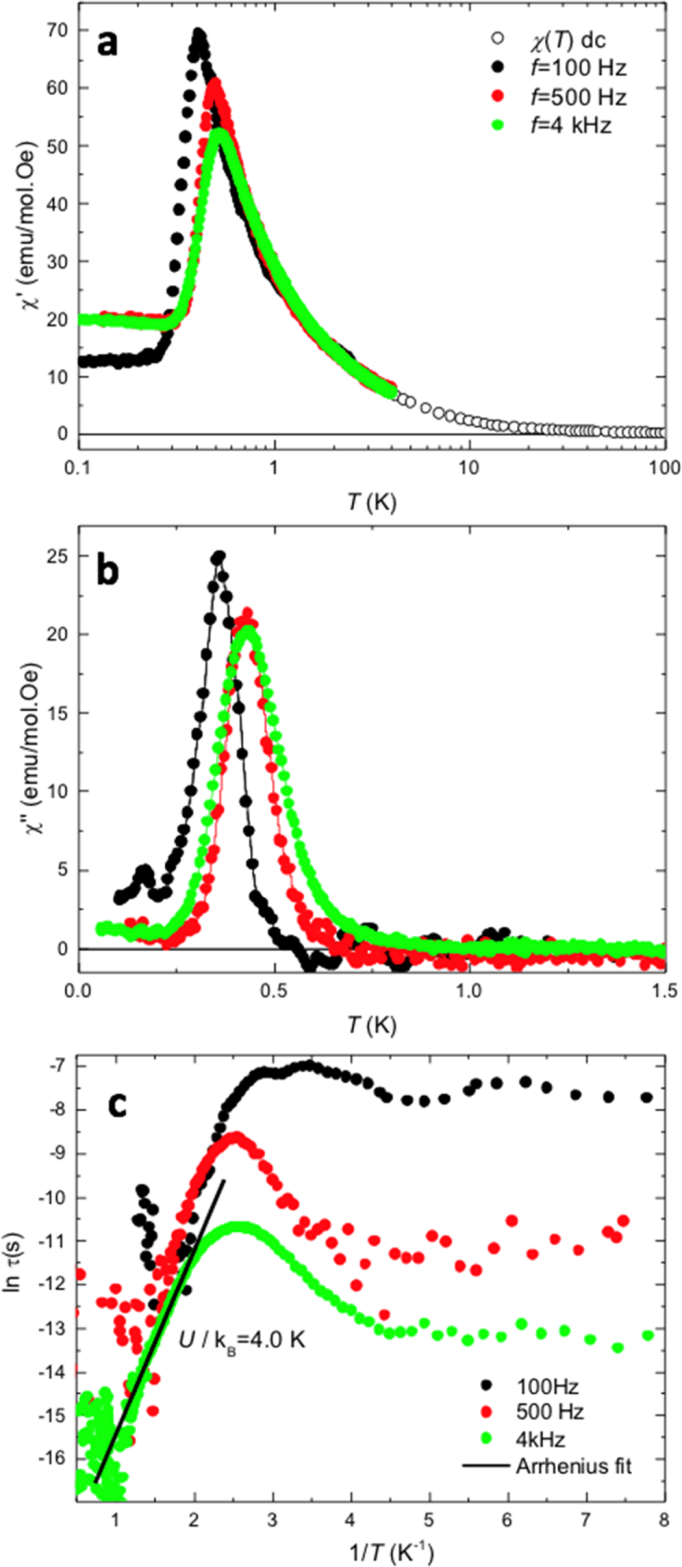
Ac susceptibility. (a) In-phase χ′(*T*, *f*) and (b) out-of-phase χ″(*T*, *f*) components of the susceptibility as a function of the temperature measured at three different frequencies, *f* = 100, 500, 4000 Hz; (c) dependence of the relaxation time with the inverse temperature and Arrhenius fit.

To assess the Single-Molecule Magnet dynamic behavior of {Cr_10_}, *ac* susceptibility measurements down to very low temperatures (mK) were performed in a setup equipped with a dilution refrigerator (DR), on a powder sample. Figure 9 shows the real and imaginary components of the susceptibility, χ′(*T*, *f*) and χ″(*T*, *f*), as a function of the temperature at three different frequencies (*f* = 100, 500, and 4000 Hz). The data were scaled by measuring the susceptibility by a standard SQUID in a common range of temperatures, between 1.8 and 4.5 K. A clear shift of the out-of-phase susceptibility with the frequency is observed.

The relaxation time as a function of temperature, τ (*T*), was estimated assuming a single Debye process
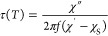
11where χ_*s*_ is the adiabatic susceptibility (i.e., in the limit of *f* → ∞), which was approximated to χ_s_ ≈ 0, and ω = 2π*f* fulfills the condition ωτ ≪1. Figure 9 shows the τ(*T*) dependence obtained for the three measured frequencies. Left aside the vertical shift between the data at the three different frequencies, the relaxation time affords a fit to an Arrhenius-like dependence, τ(*T*) = τ_0_exp(*U*/*k*_B_*T*), with an activation energy *U*/*k*_B_ = 4.0 ± 0.5 K, between ∼1.1 and 2 K. This value is in good agreement with the value expected for an SMM with a ground state *S* = 9, *S*_z_ = ± 9, and anisotropy *D*/*k*_B_ = −0.045(2) K, *U*/*k*_B_ = (*D*/*k*_B_)*S*_*z*_^2^ = 3.7(2) K.

Finally, it is interesting to address the entanglement mechanism in the {Cr_10_} wheel, as an important asset for the application of these SMMs in quantum information technologies and for the interest in investigating quantum phenomena.^3^ Indeed, homometallic {Cr_8_} wheels and heterometallic {Cr_7_M} wheels have been the subject of numerous theoretical and experimental studies on different kinds of entanglement.^67−69^ The {Cr_10_} case here presented is relevant, as it is essentially different from the previous homo- and heteronuclear-nuclear wheels previously considered in the literature, where AF interactions were dominant.

Experimental determination of thermal entanglement in spin clusters is possible with the use of entanglement witnesses (EW) macroscopic observables which allow us to discriminate between fully separable and entangled spin states. Magnetic susceptibility is a macroscopic EW,^70^ which depends on the correlations of all of the spin pairs. In this reference, it is shown that a system consisting of *N* spins *S*_i_ is in an entangled state when the inequality of the reduced averaged components of the magnetic susceptibility fulfills the condition: χ®^*x*^ + χ®^*y*^ + χ®^*z*^ < *NS*_*i*_/*k*_B_*T*. This criterion was applied to test the entanglement state in clusters of Na_2_Cu_5_Si_4_O_14,_^71^ where the inequality was written as: χ^exp^ = (χ®^*x*^ + χ®^*y*^ + χ®^*z*^)/3 < (g^2^ μ_B_^2^)*NS*_i_/3*k*_B_*T*.

In our case, we deal with a cluster of 10 spins *S*_i_, and knowing the powder susceptibility per mol, χ^powder^ = (χ^*x*^ + χ^*y*^ + χ^*z*^)/3 and *N* = *N*_A_(10*S*_i_), where *N*_A_ is the Avogadro’s number, the entanglement witness parameter may be introduced as


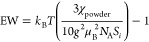
12where *S*_i_ = 3/2 is the Cr spin. Thus, the systems is in the entangled state if EW < 0 (although it must be noted that EW ≥ 0 does not necessarily implies separability).

**Figure 10:**
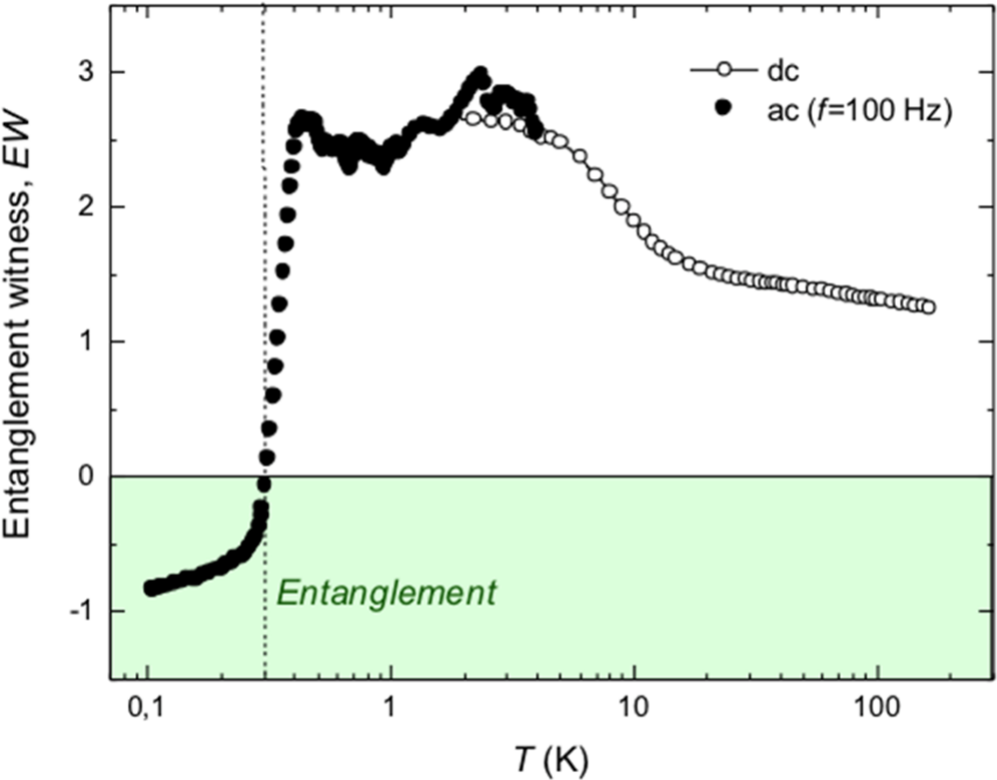
Entanglement witness, EW, as a function of the temperature measured for the powder {Cr_10_} sample from dc SQUID measurement (*T* = 1.8–300 K) and *ac* susceptibility measurements at low frequency, *f* = 100 Hz (*T* = 0.1–4 K). Entanglement is observed for *T* < 0.3 K.

Figure 10 shows the EW determined in the range of temperatures between 300 K and 0.1 K combining *dc* and *ac* susceptibility measurements at the lowest frequency (100 Hz). It is observed that {Cr_10_} exhibits entanglement (EW < 0) at very low temperatures, below 0.3 K.

In the absence of or negligible intercluster interaction, we may assume to be dealing with multispin entanglement of independent spin clusters. Troiani and Siloi^67^ have developed a general approach for deriving the degree of multipartite entanglement in one-dimensional spin systems, for S *>* 1/2 spins, where multispin entanglement is limited to *T* ≲ *J*/*k*_B_. Therefore, our result is fully compatible with theoretical predictions of multispin entanglement, resulting in an experimental realization of k-spin entanglement in a solid-state system. Hence, it may be interesting to pursue this new concept further, in {Cr_10_} or related compounds, for applications in quantum information.

## Conclusions

The magnetic properties of the molecular wheel {Cr_10_}, consisting of 10 Cr^3+^ ions with *S*_i_ = 3/2, have been studied in-depth. The magnetization at low temperatures shows anisotropy, which can be well explained by the Hamiltonian for the isolated molecules with total spin *S* = 9 and uniaxial anisotropy *D*/*k*_B_ = *–*0.045 K with the easy axis perpendicular to the {Cr_10_} molecule mean plane, in agreement with previous EPR results.

The origin of this unusual intermediate *S* = 9 is here disclosed. The temperature, magnetic field, and angular dependencies of the magnetization have been rationalized within a magnetic model, including the anisotropy, and a coupling scheme in which {Cr_10_} consists of two semicrowns containing four Cr ions with FM coupling (*J*_FM_/k_B_ = 2.0 K), separated by two Cr ions with asymmetric AF coupling (*J*_23_/*k*_B_ = *J*_78_/*k*_B_ = −2.0 K; *J*_34_/*k*_B_ = *J*_89_/*k*_B_ = −0.25 K).

The INS experiments allowed us to confirm this microscopic model and the values of the anisotropy and the exchange interactions. Other sets of *J*_ij_ and *D*_i_ parameters were incompatible with INS data. The transitions observed were in good correspondence with the predicted Δ*S* = 0 and Δ*S* = 1 transitions for an *S* = 9 ground state, next excited *S* = 8 multiplet, and *S* = 10 multiplets lying at higher energies. This result can be compared to that earlier proposed to interpret the INS spectra of the compound [Cr_10_(OMe)_20_(O_2_CMe)_10_] (**1**) of the same wheel family.^36^ The INS spectrum was qualitatively similar, but the peaks differed strongly in energy. The ground state in that case was found to be an *S* = 15 multiplet, corresponding to a totally different spin scheme, including at least two different *J*_FM_ interactions in the wheel. Thus, the {Cr_10_} studied here is very different, having an intermediate spin *S* = 9 ground state which is not as high as the potential total spin *S* = 15 if all of the exchange interactions were FM.

In the absence of magnetic field, the anisotropy stabilizes the degenerate spin states of *S*_z_ = ±9 at low temperatures, which correspond to opposite directions of the magnetization. SMM behavior is observed with an activation energy *U*/k_B_ = 4.0(5) K in very good agreement with the expected value for a cluster with total spin S = 9 and magnetic uniaxial anisotropy *D*, *U*/k_B_ = (*D*/k_B_)*S*_*z*_^2^ = 3.7(2) K. The SMM behavior of {Cr_10_} has a resemblance with Fe_4_, Fe_8_, Mn_12_ clusters.

Our experimental results, supported by *ab initio* and DFT calculations, show that the anisotropy, as well as the exchange values and the rupture of symmetry in the interactions in the {Cr_10_} molecule, are associated with small (∼ 1%) differences in the angles between the alkoxy Cr–O–Cr angles connecting the Cr ions. The earlier study of the family of Cr^3+^ wheels [Cr_10_(OR)_20_(O_2_CR’)_10_] in powder samples reported by Low et al.,^36^ already revealed the large dependence of the magnetic behavior on the type of ligand. Fraser et al.^50^ ascribed the occurrence of predominant ferromagnetic Cr–Cr interactions in carboxylate/alkoxide bridging units, as used in {Cr_10_}, to the peculiar orbital counter-complementarity effect introduced by the carboxylate, and showed through the systematic magneto-structural study of differently coordinated Cr_2_ dimers that small distortions can give rise to substantial variations in the Cr–Cr exchange coupling, even changing the sign. In the present work, we have shown how such small distortions are able to originate drastic changes in the magnetic behavior of a large cyclic cluster and that the multispin approach is mandatory to tackle this problem.

Maximization of the ground state (*S* = 15) would require the use of carboxylate/alkoxide units with FM coupling less sensitive to structural changes. The results of DFT calculations performed on a family of dinuclear [Cr_2_(Me-deaH)_2_(O_2_CH)Cl_2_]Cl^50^ compounds suggest the combination of carboxylate- and diethanolamine-type ligands may be useful for that purpose.

## Methods

### Samples

{Cr_10_(OMe)_20_(O_2_CCMe_3_)_10_} single crystals were prepared by a solvothermal reaction similar to that previously described^36^ but using {[Cr_6_F_7_(O_2_CCMe_3_)_10_][NH_2_Et_3_]_3_}_2_^72,73^ as {Cr_10_} wheel precursor. The crystal structure was checked by X-ray diffraction and coincided with that described earlier.^36^ The {Cr_10_} cyclic structure is formed by 10 Cr^3+^ ions nearly lying on an equatorial plane. The ring diameter is about 9.7 Å. Each pair of Cr–Cr ions is bridged by a μ_2_-carboxylate and two μ_2_-alkoxides. The alkoxides between n.n. Cr^3+^ ions point toward and away from the wheel, with one lying above and the other below the ring’s plane (Figure 1). The crystal unit cell is triclinic (space group (SG) *P*1̅ *Z* = 1) with parameters *a* = 9.867 Å, *b* = 16.990 Å, *c* = 17.898 Å, α = 115.098°, β = 99.908°, γ = 97.185^o^. Within the crystal, the molecules pack in columns, with the stacking direction parallel to the *a*-axis.

### Dc Magnetometry

Magnetization and *dc* susceptibility measurements in the temperature range of 1.8–298 K in applied magnetic fields up to 50 kOe were performed using a Quantum Design MPMS SQUID magnetometer. Experiments were conducted on polycrystalline samples, fixed in cotton to prevent grain orientation. Crushing the sample into a powder, or embedding it in n-hexane provoked changes in the magnetization *M*(*H*) curve (see S1).

### Heat Capacity

Heat capacity as a function of the temperature down to 0.3 K was measured at zero applied field on a pressed powder pellet fixed with Apiezon N grease, using a Quantum Design PPMS equipped with a ^3^He refrigerator.

### Angular Magnetometry

Additional magnetometry measurements as a function of the angle were performed on a single crystal (SC) to study the magnetic anisotropy. The SC is triclinic, with space group **P1̅** and a single molecule per f.u.; thus, all {Cr_10_} molecules are parallel to each other. The normal axis perpendicular to the wheel makes an angle of 34.784° with the crystallographic axis *a*, 121.105° with *b*, and 67.127° with *c*.^74^ The size of the crystals as grown is typically 1 mm × 0.5 mm × 0.5 mm. A single crystal of 26 μg was oriented by means of X-ray single-crystal diffraction and glued to a sapphire plate with the axis perpendicular to the average plane of the {Cr_10_} wheels perpendicular to it. The sapphire plate was placed on the rotator plate of the magnetometer. Two experiments were carried out: (i) rotating the sample around an axis contained within the Cr_10_ plane, and (ii) around the molecule normal axis, parallel to the {Cr_10_} quasi-fivefold symmetry axis. The measured magnetic moments were corrected for the diamagnetic backgrounds of the sapphire and glue.

### Ac Susceptibility

*Ac* susceptibility measurements in the range between 0.1 and 5.5 K, at *H*_ac_ = 4.1 Oe, *H*_dc_ = 0, and three different frequencies, *f* = 100, 500, and 4000 Hz, were performed on a powder sample compacted into a pellet using a dilution refrigerator-based susceptometer at Quantum Design San Diego.

### XANES and XMCD

X-ray absorption near-edge structure (XANES) and X-ray magnetic circular dichroism measurements (XMCD) at the Cr K-edge (5989.2 eV) were performed at the ESRF ID12 beamline. A pellet sample was formed by mixing the {Cr_10_} powder with graphite powder, to increase thermal conductivity. The Apple-II undulator and a double-Si(111) crystal monocromator were used to collect the spectra at the respective energies. All XANES spectra were recorded using total fluorescence yield detection mode in backscattering geometry and were subsequently corrected for reabsorption effects. Polarization of the circular light was over 84%. XMCD was obtained by differences of two consecutive XANES spectra measured with opposite photon helicities at a fixed magnetic field value (170 kOe), orienting the field in two inverse directions to ensure the absence of experimental artifacts. The spectra were taken at a temperature of *T* = 7.5 K.

### Inelastic Neutron Scattering (INS)

Measurements were carried out on a 0.7 g of a nondeuterated fresh powder sample on the LET cold neutron multichopper time-of-flight spectrometer at the ISIS Facility, U.K.^75^ The instrument operates in repetition rate multiplication mode giving simultaneous multiple incident energies. In the present case, the incident energy was set at *E*_i_ = 1.5 meV with chopper 5 operating at 200 Hz and the pulse remover chopper 3 at 100 Hz. This configuration allows the simultaneous collection of data with *E*_i_ = 15, 5.1, 2.5, and 1.5 meV. The sample was inserted into a cryostat cooled down to a base temperature of 1.8 K. Measurements were also taken at 5 and 10 K. The scattering function *S*(*Q*, ℏω) was measured as a function of the transferred energy ω = *E*_i_ – *E*_f_ and transferred momentum *Q* = |*Q*| (where *E*_i_ and *E*_f_ are the energies of the incident and scattered neutrons, respectively, and ***Q*** = ***k_i_*** – ***k_f_***). The sample consisted of powder obtained from single crystals. Since the sample was not deuterated, it contained a large number of hydrogen atoms, which would contribute strongly to the incoherent neutron scattering. However, there exists a window of transfer energies from ca. 0.1 to 3 meV with a rather small hydrogen scattering, which makes possible such an experiment.^64^

### *Ab Initio* and DFT Calculations

*Ab initio* calculations using the ORCA 4.0 software^76,77^ were performed to identify an appropriate magnetic model: the single-ion magnetic anisotropies and their main axes and gyromagnetic factors were studied by the CASSCF/NEVPT2 method,^78,79^ in which spin–orbit coupling and spin–spin coupling relativistic effects, which are at the origin of the magnetic anisotropy, are included *a posteriori*. In addition, the nature of the magnetic interactions between neighbor Cr^3+^ ions was explored by the DFT broken symmetry method as formulated by Yamaguchi.^79^ The CASSCF/NEVPT2 calculations were done on a cluster of atoms containing the studied Cr^3+^ ion, its two neighbor Cr^3+^ ions, replaced by diamagnetic Ga^3+^ ions, and all its surrounding ligands. The cluster also included the second neighbor Cr^3+^ ion replaced by Mg^+2^. The basis set was the DKH-Def2-TZVP^80^ for all of the atoms, which incorporates scalar relativistic effects. To speed up the calculations, the SARC/J auxiliary basis^80^ along with the resolution of identity (RI)^81^ and the chain-of-spheres (COSX) approximations^82^ were used. In the CASSCF calculations, the active space consisted of 10 Cr^3+^ 3d and 3d′ orbitals containing three electrons (CASSCF(3,10)). The state-averaged CASSCF calculation included 10 quartets. Then, the NEVPT2 calculations were performed with the CASSCF(3,10) reference space for the treatment of the dynamical correlation energy. After that, the effect of the spin–orbit coupling was taken into account using a mean-field operator (SOMF),^83,84^ which was diagonalized on the basis of the previous CASSCF wavefunctions. As for the DFT calculations, they were performed on a cluster of atoms containing the two Cr^3+^ ions involved in the studied interaction and its two neighbor Cr^3+^ ions, these last ones replaced by diamagnetic Ga^3+^ ions. The cluster also included the ligands connecting the four Cr^3+^ ions and F^-^ ions replacing the ligands of the two external Cr^3+^ ions not connected with the central Cr^3+^ ions. The basis set was the Def2-TZVP^85^ for all of the atoms. To speed up the calculations, the Def2/J auxiliary basis^85^ along with the resolution of identity (RI) and the chain-of-spheres (COSX) approximations were used. The employed exchange-correlation functional was the B3LYP^86^ hybrid one, whereas the accuracy of the integration grid was increased with respect to the default values using the ORCA parameters Grid5 and FinalGrid6.

## Supporting Information Available

Magnetization measurements on bulk (S1); XMCD of {Cr_10_} (S2); *ab initio* calculations (S3); DFT calculation of interactions (S4); effective anisotropy tensor (S5); and energy levels for the isotropic and anisotropic Hamiltonian (S6) (PDF)
