# Supplementary material for: Origin of the Unusual Ground-State Spin S = 9 in a Cr10 Single-Molecule Magnet
Source: J Am Chem Soc. 2022 Jun 27;144(27):12520–35. doi: 10.1021/jacs.2c05453 (PMC9979690; doi:10.1021/jacs.2c05453)
Supplement: Supplementary file 1 — ja2c05453_si_001.pdf [file ja2c05453_si_001.pdf]

Supplementary Information

Origin of the unusual ground state spin S=9 in a Cr_{10} Single-Molecule Magnet

Javier Rubín[1,2*], Ana Arauzo[1,3,4], Elena Bartolomé[5*], Francesco Sedona[6], Marzio Rancan[7], Lidia Armelao[6,8], Javier Luzón[9], Tatiana Guidi[10,11], Elena Garlatti[12], Fabrice Wilhelm[13], Andrei Rogalev[13], Andreas Amann[14], Stefano Spagna[14], Juan Bartolomé[1,4], Fernando Bartolomé[1,4*]

[1] Instituto de Nanociencia y Materiales de Aragón (INMA), CSIC-Universidad de Zaragoza, Zaragoza 50009, Spain
[2] Departamento de Ciencia y Tecnología de Materiales y Fluidos, Universidad de Zaragoza, 50018 Zaragoza, Spain
[3] Servicio de Medidas Físicas, Universidad de Zaragoza, Pedro Cerbuna 12, 50009 Zaragoza, Spain
[4] Departamento de Física de la Materia Condensada, Universidad de Zaragoza, Zaragoza 50009, Spain
[5] Escola Universitària Salesiana de Sarrià (EUSS), Passeig Sant Joan Bosco 74, 08017 Barcelona, Spain
[6] Dipartimento di Scienze Chimiche, Università di Padova, Via Marzolo 1, 35131 Padova, Italy.
[7] Institute of Condensed Matter Chemistry and Technologies for Energy (ICMATE), National Research Council (CNR), c/o Department of Chemistry, University of Padova, via F. Marzolo 1, 35131 Padova, Italy.
[8] Department of Chemical Sciences and Materials Technologies (DSCTM), National Research Council (CNR), Piazzale A. Moro 7, 00185 Roma, Italy.
[9] Centro Universitario de la Defensa. Academia General Militar, Zaragoza, Spain.
[10] Physics Division, School of Science and Technology, University of Camerino, Via Madonna delle Carceri 9, Camerino, MC 62032, Italy.
[11] ISIS facility, Rutherford Appleton Laboratory, Chilton, Didcot, OX11 0QX Oxfordshire, UK.
[12] Dipartimento di Science Matematiche, Fisiche e Informatiche, Università di Parma, Parco Area delle Scienze 7/A, 43124 Parma, Italy.
[13] ESRF - The European Synchrotron Radiation Facility, 71 Avenue des Martyrs CS40220, F-38043 Grenoble Cedex 09, France.
[14] Quantum Design Inc., San Diego, CA 92121, USA.
Email: jrubin@unizar.es; bartolome@unizar.es; ebartolome@euss.es.

S1. Magnetization measurements on bulk

S2. XMCD of {Cr_{10}}

S3. *Ab initio* calculations

S4. DFT calculation of interactions

S5. Effective anisotropy tensor

S6. Energy levels for the isotropic and anisotropic Hamiltonian

S1. Magnetization measurements on bulk

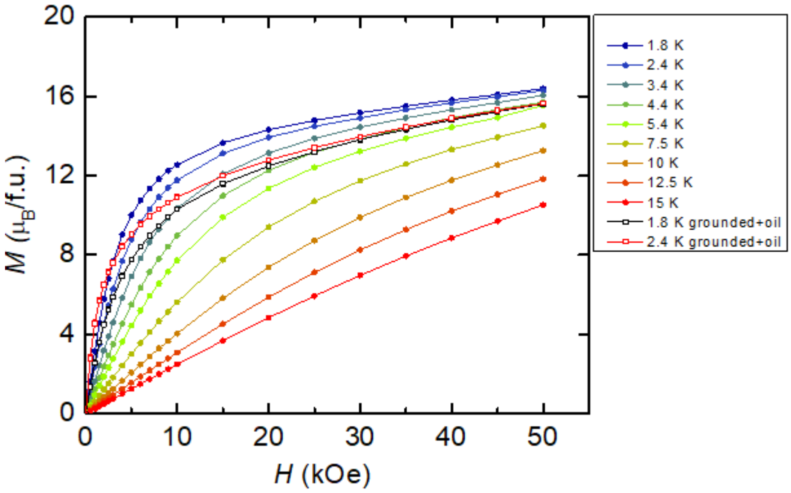

Figure S1.1. Magnetization as a function of the applied magnetic field for a {Cr_{10}} powder sample, at different temperatures. The sample was not grounded or mixed with oil, it was only fixed in cotton. For comparison sake, the *M*(*H*) curves measured at *T*=1.8 K and *T*=2.4 K for a powder sample from the same batch, grounded on a mortar and mixed with oil, are shown.

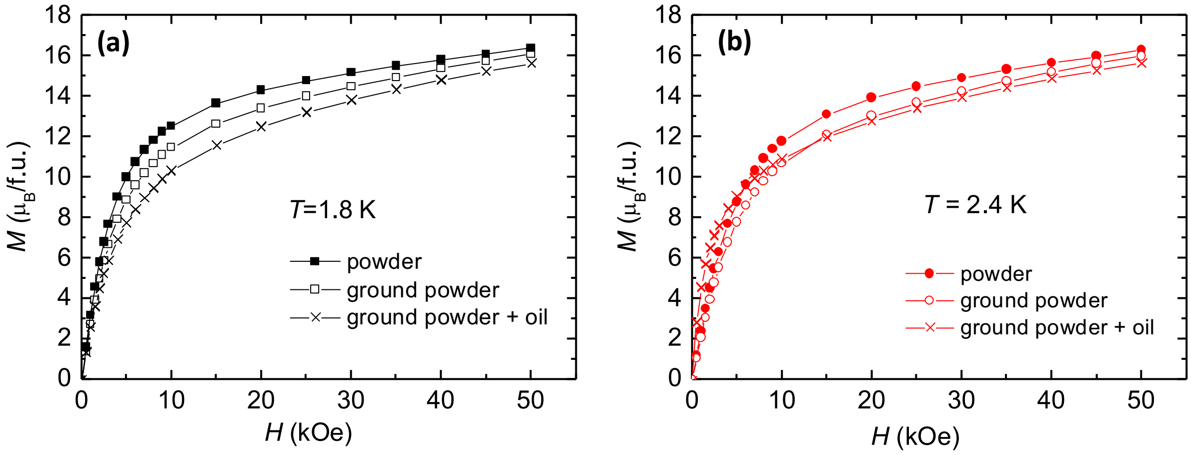

Figure S1.2. Magnetization as a function of the applied magnetic field for a powder sample of {Cr_{10}}, the same sample ground on a mortar, and the ground powder embedded in oil, at *T*=1.8 K (a) and *T*=2.4 K (b).

S2. XMCD of {Cr_{10}}

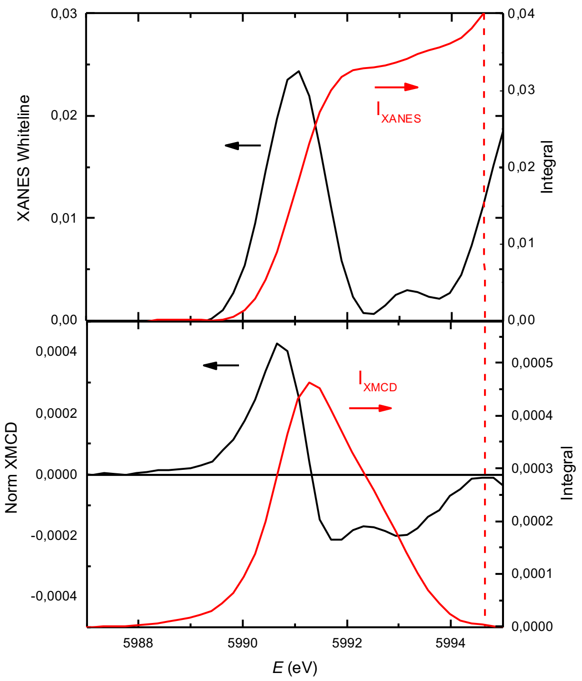

Figure S2.1. XANES and XMCD at Cr K pre-edge. Top: Left scale, background-subtracted normalized x-ray absorption spectroscopy (XANES whiteline); Right scale: whiteline integral, $(I_{XANES})_{pre\text{-}edge}$. Bottom: Left scale, normalized x-ray magnetic circular dichroism (XMCD); Right scale: integrated XMCD, $(I_{XMCD})_{pre\text{-}edge}$.

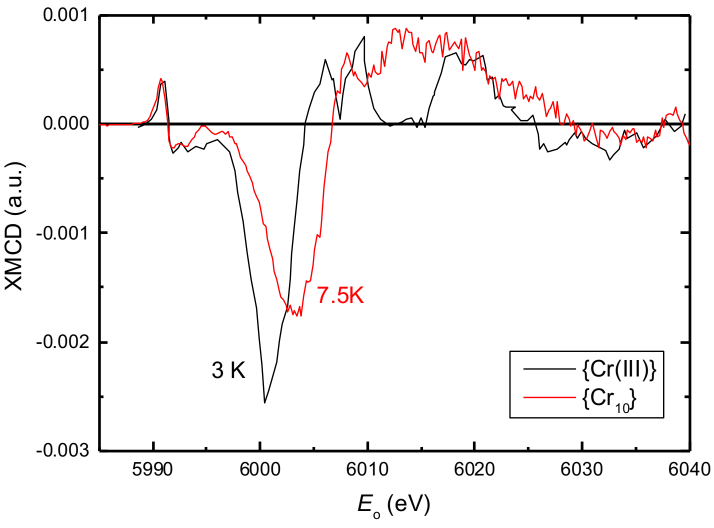

Figure S2.2. Comparison of the XMCD at the Cr K-edge of the *trans*-[Cr(III)Cl_2(pyridine)$_4$](ClO_4).1/4H_2O, Cr(III) compound, abbreviated {Cr(III)},[5] measured at *T*=3 K and *H*=170 kOe (black), and of {Cr_{10}} at *T*=7.5 K and *H*=170 kOe. The magnetic moment of {Cr(III)} is saturated at 3 K and 170 kOe, and amounts to 3.1 μ_B.[5] The intensity of the XMCD peak of {Cr(III)} (I_{XMCD}= -2.48 x 10^{-3}) needs to be multiplied by a factor 0.68 to scale with that of {Cr_{10}} (I_{XMCD}=-1.69 x 10^{-3}), yielding a value of m_{Cr}=2.1 μ_B per Cr ion, at *T* = 7.5 K and 170 kOe for {Cr_{10}}.

S3. *Ab initio* calculations

The computation of the magnetic anisotropy of the Cr ions in the {Cr_{10}} wheel was done using molecular clusters containing the studied Cr^{3+} ion and the four closest Cr neighbors in addition to the ligands connecting these five Cr^{3+} ions. Actually, the ligand moieties far from the studied Cr ion were simplified in order to reduce the computation time and the neighbor Cr^{3+} ions were replaced by Ga^{3+} the closest ones and Mg^{2+} the second closest ones in order to use closed-shell ions and have an almost neutrally charged molecular cluster.

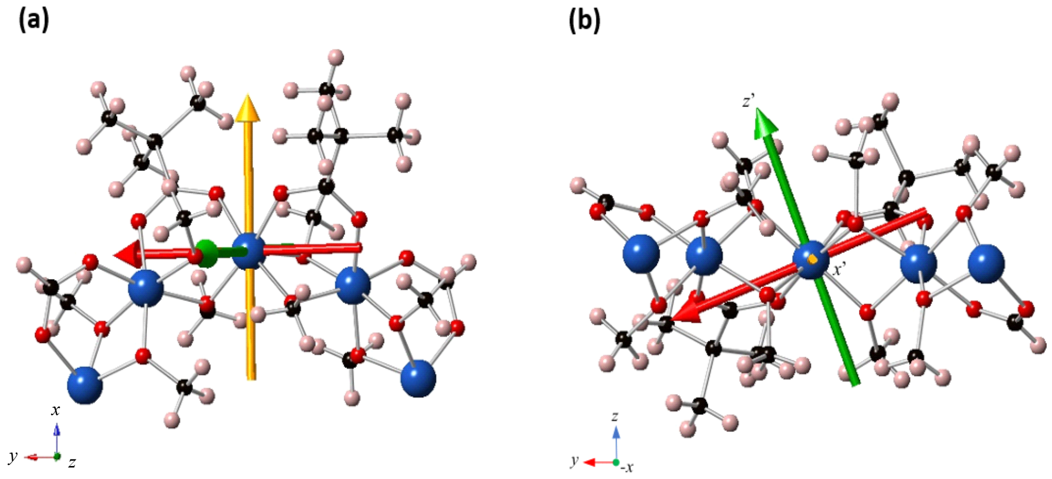

Figure S3.1. Molecular cluster, local axes xyz for the *ab initio* computation, and principal axes *x'y'z'* of the derived anisotropy tensor for the ion Cr3. (a) planar *xy*-view, (b) lateral, *yz*-view. Green: easy axis (EA) of magnetization; Red: hard axis (HA) of magnetization.

Figure S3.1 shows one of the five employed molecular clusters and the local axes employed in the computation: *x*-axis along the radial direction of the wheel, *y*-axis tangential to the {Cr_{10}} wheel, and *z*-axis perpendicular to the plane defined by the wheel.

For the five different Cr^{3+} ions, the *ab initio* calculations allowed to obtain the E_i and D_i values of a single-ion zero-field splitting Hamiltonian with axial and tetragonal terms:

$$\mathcal{H}_{ZFS} = D_i S_{z'}^2 + E_i(S_{x'}^2 - S_{y'}^2),$$

where S, $S_{x'}$, $S_{y'}$ and $S_{z'}$ are spin and its components for the Cr^{3+} ion, and *x'*, *y'* and *z'* are the principal axes of the anisotropy tensor, i.e., those for which the tensor is diagonal:

$$\hat{D} = \begin{pmatrix} D_{x'x'} & 0 & 0 \\ 0 & D_{y'y'} & 0 \\ 0 & 0 & D_{z'z'} \end{pmatrix} = \begin{pmatrix} \left(-\frac{1}{3}+\frac{E}{D}\right)D & 0 & 0 \\ 0 & \left(-\frac{1}{3}-\frac{E}{D}\right)D & 0 \\ 0 & 0 & \frac{2}{3}D \end{pmatrix}.$$

The calculated D_i and E_i/D_i values for the five different Cr^{3+} ions are shown in Table S3.1, while the cosine directors of the single-ion anisotropy principal axes with respect to the local axes are given in Table S3.2.

Table S3.1. *Ab initio* calculated magnetic anisotropy parameters of the Cr ions in Cr_{10} wheel.

Cr ion	D_i/k_B (K)	E_i / D_i
Cr1	–0.32	0.33
Cr2	–0.25	0.28
Cr3	–0.20	0.25
Cr4	–0.27	0.26
Cr5	–0.33	0.33

For all five Cr^{3+} ions shown $D_i < 0$ and the E_i/D_i values are close to 1/3, which indicates a system midway between uniaxial and planar magnetic anisotropy. For all the Cr^{3+} ions the direction of the uniaxial magnetic anisotropy axes *z'*, is close to the *z* local axis (perpendicular to the

wheel), whereas the HA is close to the *y* local axis (in the tangencial direction of the wheel). A representation of this set of anisotropy axes is displayed in Fig. S3.1 for Cr3 ion.

Table S3.2. Cosine directors of the single-ion anisotropy principal axes with respect to the local axes.

Principal axis	Cr ion	Components in the local axes		
		x	*y*	*z*
x′	Cr1	0.026	0.903	0.429
	Cr2	0.196	0.919	–0.342
	Cr3	–0.025	0.964	–0.264
	Cr4	–0.145	0.936	0.321
	Cr5	–0.015	0.922	–0.387
y′	Cr1	0.995	0.020	0.102
	Cr2	0.981	–0.178	0.084
	Cr3	0.994	–0.002	–0.106
	Cr4	0.987	–0.114	0.114
	Cr5	0.999	0.027	0.025
z′	Cr1	–0.101	0.429	0.898
	Cr2	–0.016	0.351	0.936
	Cr3	0.103	0.265	0.959
	Cr4	–0.070	0.333	0.940
	Cr5	–0.033	0.387	0.922

The easy axes of the magnetization (EAM) calculated in this section are shown in Figs. 6a and 6b.

S4. DFT calculation of interactions

See Methods Section in Main text.

distance	d(Å)	Cr ion	D_i(K)	E_i/D_i ratio	Angle	Interior (°)	Exterior (°)	Interaction	J_{ij}/k_B (K)
**Cr1-Cr1i**	9.527	**Cr1**	0.32	0.33	**Cr1-O-Cr2**	98.25	98.87	**Cr1-Cr2**	7.1
**Cr2-Cr2i**	9.567	**Cr2**	0.25	0.28	**Cr2-O-Cr3**	99.29	98.01	**Cr2-Cr3**	-0.82
**Cr5-Cr5i**	9.582	**Cr3**	0.20	0.25	**Cr3-O-Cr4**	99.08	98.36	**Cr3-Cr4**	0.29
**Cr3-Cr3i**	9.656	**Cr4**	0.27	0.26	**Cr4-O-Cr5**	98.49	98.67	**Cr4-Cr5**	8.56
**Cr4-Cr4i**	9.668	**Cr5**	0.33	0.33	**Cr5-O-Cr1**	98.08	98.99	**Cr5-Cr1i**	12.2

Table S4. DFT calculated Cr-Cr magnetic interactions.

The deviation angle of the anisotropy axis of each Cr_i ion with respect to the vertical axis of the molecule (z-axis), perpendicular to the $\{Cr_{10}\}$ plane have been calculated, from the *ab initio*-calculated cosine directors of the single-ion anisotropy principal axes with respect to the local axes, given in Table S3.2. Figure S4.1 shows that the deviation angles are correlated with the exchange interaction J_{ij} between each pair of Cr-O-Cr ions. This stems from the fact that both depend on the local structural details around each Cr_i. The minimum deviation angle (~17°) and largest AF exchange corresponds to Cr3, for which the difference between the Cr-O-Cr angle of the inner and outer bridge is maximum and positive, whereas the largest deviation (26°) corresponds to Cr1, with the largest FM and maximum negative difference between the angles of the inner and outer Cr-O-Cr bridges.

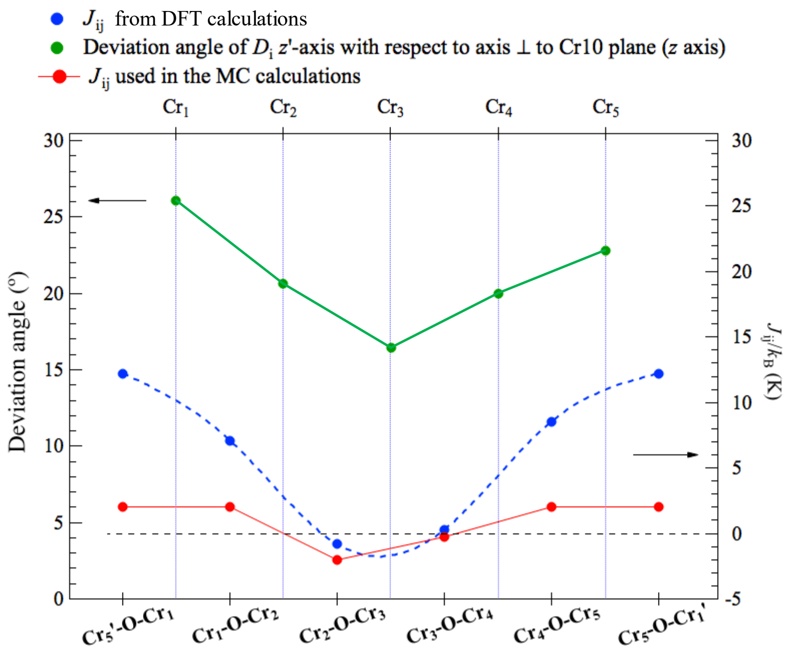

Figure S4.1. Deviation angle of the single-ion anisotropy axis of each Cr_i ion with respect to the cluster z-axis, perpendicular to the $\{Cr_{10}\}$ plane; and coupling interaction J_{ij} between each pair of Cr-O-Cr ions obtained from DFT calculations and used in MC calculations to fit the experimental magnetometry data.

S5. Effective Anisotropy Tensor

The transformation from the local anisotropy of Cr^{3+} ions, D_i , and inter-ion interaction anisotropy, D_{ij} ,to an equivalent effective anisotropy of the {Cr_{10}} wheel, D ,can be carried out in the strong isotropic exchange limit by means of a linear combination of the former anisotropies, with d_i and d_{ij} coefficients which may be calculated taking into account symmetry relations. Then, according to Ref. [1], the following relations are fulfilled

$$\widehat{D} = \widehat{D}_a + \widehat{D}_{int} = \sum_i d_i \widehat{D}_i + \sum_{i,j} d_{ij} \widehat{D}_{ij}, \qquad (1)$$

$$\sum_i d_i + 2\sum_{i,j} d_{ij} = 1, \qquad (2)$$

where $\widehat{D}_a$ reflects the anisotropy of the $\widehat{D}_i$ local anisotropy tensors caused by ligand field interactions, $\widehat{D}_{int}$ sums the contributions due to the $\widehat{D}_{ij}$ tensors caused by inter-ion anisotropy terms as, e.g., dipolar and exchange interactions, and $\widehat{D}$ is the anisotropy tensor of the full molecule. This method has been successful in several analyses of dinuclear cluster anisotropy[2,3,4].

The coefficients d_i and d_{ij} are calculated assuming colinear orientation of spins which constitute the {Cr_{10}} wheel, i.e. two semi-wheels, each with four S=3/2 Cr^{3+} spins coupled FM, separated by two Cr^{3+} ions AF coupled asymmetrically. We have calculated the coefficients d_i following the iteration formulae given in Ref. 1. Under the assumption that only n.n. interactions are relevant, $\sum_{i,j} d_{ij} = \sum_{i=1}^{N} d_{i,i+1} = 10 d_{CrCr}$, where d_{CrCr} is the average value of $d_{i,i+1}$. Substituting the d_i coefficients in Eq. 2, the value of d_{CrCr}=0.0428 coefficient is obtained. The coefficients are collected in Table S5.1.

Once the d_i are calculated, we proceed to calculate the $\widehat{D}_a = \sum_i d_i \widehat{D}_i$ tensor. In fact, the tensors $\widehat{D}_i$ must be written in a common coordinate system, giving rise to a non-diagonal molecular tensor which has to be diagonalized to obtain the equivalent anisotropy constants D_a and E_a, from single-ion anisotropies, i.e. excluding inter-ion interactions. The result is D_a/k_B = –0.0332 K and E_a/k_B = –0.0048 K (E/D = 0.0145). The common coordinate system used was that of Cr3 (Fig. S3.1) and the matrix of eigenvectors of the diagonal $\widehat{D}$ tensor is:

$$\begin{pmatrix} -09737 & -0.2265 & 0.0300 \\ -0.2257 & 0.9740 & 0.0058 \\ 0.0298 & 0.0013 & 0.9995 \end{pmatrix},$$

which shows that magnetic anisotropy the {Cr_{10}} ring is uniaxial with a rhombic distortion smaller than that of the constituent Cr^{3+} ions, and the direction of this axis (z') is very close to the perpendicular to the ring's mean plane (≈2.7° out of the axis z in Fig. S3.1).

d_1	d_2	d_3	d_4	d_5	d_6	d_7	d_8	d_9	d_{10}	d_{CrCr}
0.00862	0.00862	0.00359	0.01796	0.01796	0.01796	0.01796	0.01233	0.01961	0.01961	0.0428

Table S5. Coefficients d_i calculated in a collinear arrangement of spins locating the antiparallel spins on Cr3 and its equivalent site by inversion. The d_{CrCr} coefficient is calculated applying Eq. 2 .

The difference between the experimental value D/k_B=-0.045(2) K and the calculated value D_a/k_B=-0.033 K may be ascribed to the uncertainty originated by the approximations applied in the *ab initio* methods or the deviation from coliniarity. But it could also be due to a contribution from the inter-ion interaction contribution, D_{int} term in Eq. 1. To estimate it we substitute each D_{ij} by an average value D_{CrCr}. Since we have evaluated the d_{CrCr} above, by substitution in Eq. 1.

$$D_{CrCr} = \frac{D - D_a}{10 \times d_{CrCr}} \qquad (3)$$

The value D_{CrCr}/k_B=-0.028 K is obtained. This estimation yields an upper limit in the possible contribution to the effective anisotropy by inter-ion interaction anisotopy. In any case, its effect is to reinforce the uniaxial anisotropy perpendicular to the wheel plane.

S6. Energy levels for the isotropic and anisotropic Hamiltonian

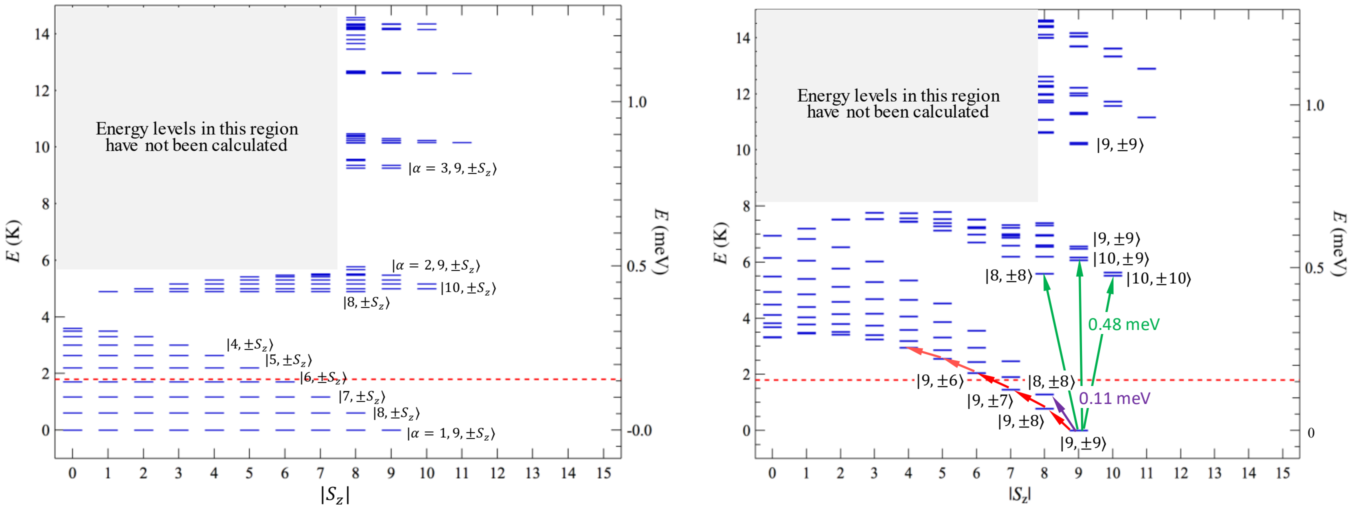

Figure S6. Energy levels scheme calculated with the Hamiltonian eq. (6) in the main text with $D_i = 0\ \forall i$ (left figure, isotropic case)) and $D_i \neq 0$ (right figure), plotted as a function of S_z with respect to the lowest energy found in each case. The states are fully labelled as $|\alpha, S, S_z\rangle$ where α denotes different configurations of the 10 single-ion spins to produce the ring's total spin S and third component S_z. The full labelling is shown only for $S = 9$ in the isotropic case for the sake of simplicity. In this representation of energy levels *vs* $|S_z|$ in the isotropic case, all $2S_z + 1$ degenerate levels are shown, which may help in tracking the levels splitting by the uniaxial anisotropy shown on the right. On the right panel, the transitions observable by INS are shown by the arrows and explained in the main text.

REFERENCES

(1) Bencini, D.; Gatteschi, A. *Electron Paramagentic Resonance of Exchange Coupled Systems*; Springer-Verlag: Berlin Heidelberg, 1990.

(2) Singh, S. K.; Rajaraman, G. Probing the Origin of Magnetic Anisotropy in a Dinuclear {MnIIICuII} Single-Molecule Magnet: The Role of Exchange Anisotropy. *Chem. Eur. J.* **2014**, *20*, 5214 – 5218.

(3) Gupta, T.; Rajaraman, G. Modelling Spin Hamiltonian Pa-Rameters of Molecular Nanomagnets. *Chem. Com-mun.,* **2016**, *52*, 8972.

(4) Vignesh, K. R.; Langley, S. K.; Gartshore, C. J.; Borilović, I.; Forsyth, C. M.; Rajaraman, G.; Murray, K. S. Rationalizing the Sign and Magnitude of the Magnetic Coupling and Anisotropy in Dinuclear Manganese(III) Complexes. *Dalt. Trans.* **2018**, *47*, 11820.

(5) Pedersen, K. S.; Perlepe, P.; Aubrey, M. L.; Woodruff, D. N.; Reyes-Lillo, S. E.; Anders Reinholdt, L.; Voigt, Z. L.; Borup, K.; Rouzières, M.; Samohvalov, D.; et al. Formation of the Layered Conductive Magnet CrCl2(Pyrazine)2 through Redox-Active Coordination Chemistry. *Nat. Chem.* **2018**, *10*, 1056–1061.
